# *De Novo* Structure Prediction of Globular Proteins Aided by Sequence Variation-Derived Contacts

**DOI:** 10.1371/journal.pone.0092197

**Published:** 2014-03-17

**Authors:** Tomasz Kosciolek, David T. Jones

**Affiliations:** 1 Bioinformatics Group, Department of Computer Science, University College London, London, United Kingdom; 2 Institute of Structural and Molecular Biology, University College London, London, United Kingdom; University Of Oxford, United Kingdom

## Abstract

The advent of high accuracy residue-residue intra-protein contact prediction methods enabled a significant boost in the quality of *de novo* structure predictions. Here, we investigate the potential benefits of combining a well-established fragment-based folding algorithm – FRAGFOLD, with PSICOV, a contact prediction method which uses sparse inverse covariance estimation to identify co-varying sites in multiple sequence alignments. Using a comprehensive set of 150 diverse globular target proteins, up to 266 amino acids in length, we are able to address the effectiveness and some limitations of such approaches to globular proteins in practice. Overall we find that using fragment assembly with both statistical potentials and predicted contacts is significantly better than either statistical potentials or contacts alone. Results show up to nearly 80% of correct predictions (TM-score ≥0.5) within analysed dataset and a mean TM-score of 0.54. Unsuccessful modelling cases emerged either from conformational sampling problems, or insufficient contact prediction accuracy. Nevertheless, a strong dependency of the quality of final models on the fraction of satisfied predicted long-range contacts was observed. This not only highlights the importance of these contacts on determining the protein fold, but also (combined with other ensemble-derived qualities) provides a powerful guide as to the choice of correct models and the global quality of the selected model. A proposed quality assessment scoring function achieves 0.93 precision and 0.77 recall for the discrimination of correct folds on our dataset of decoys. These findings suggest the approach is well-suited for blind predictions on a variety of globular proteins of unknown 3D structure, provided that enough homologous sequences are available to construct a large and accurate multiple sequence alignment for the initial contact prediction step.

## Introduction

For some time now, the importance of residue-residue contacts in protein structure prediction has been known and explored [1], [2], [3], [4]. Long range inter-residue contacts provide a constraint on the topology of a protein domain, greatly limiting the conformational space which needs to be sampled. Thus, a source of accurate predicted contact information might greatly facilitate de novo protein structure prediction accuracy, and unsurprisingly, the problem of residue contact prediction has drawn significant attention in the field. Examples of contact prediction range from distinguishing between correct and incorrect protein models [5], identifying direct residue contacts in protein-protein complexes [6] or as part of empirical force field for molecular dynamics simulations of protein folding [7]. In terms of contact prediction methodology, there has also been a significant recent degree of progress spanning simple mutual information calculations in multiple sequence alignments (MSAs) [8], statistical covariance analyses [1], [2], [3], [9], pattern recognition techniques (e.g. Support Vector Machine and neural network based approaches) [10], [11], [12], [13] to the ones based on. All of these methods have been recently comprehensively reviewed [14].

Until recently, however, accurate prediction of residue-residue contacts from sequence information has been a significant problem due to methods having a high rate of false positives [15], and the relative shortage of homologous sequences [16].The reason for the number of homologous sequences being a bottleneck is due to the fact that contacts may be inferred directly from evolutionary information by tracing correlated mutations in protein families represented by MSAs. A rationale for this is that residues mutate in tandem to maintain physicochemical properties of the pairs and thus, not to perturb the native fold of a protein [2], [17]. As the size of a family increases, the false positive rate of contact prediction falls, but there remain systematic errors which are present even when there is an abundance of sequence information. These systematic errors stem from systematic alignment errors occurring when building very large MSAs, phylogenetic biases, and most importantly, linked chains of covariance (indirect coupling effects) [9], [18].

The rapid development of high-throughput genomic sequencing has caused the sizes of many protein families to increase rapidly over the last 5 years and hence, both the number of known domain families and the sizes of these families have steadily increased. To date, there are over 12,000 well-characterized protein families (PFAM-A families) and estimates of the total number of protein domain families in nature has reached up to 200,000 [19]. This is of particular importance in contact prediction, as large MSAs of related sequences are required for satisfactory levels of accuracy.

The indirect coupling obstacle has only recently been resolved to a useful level by inferring residue pair couplings from a global maximum entropy model [7], [18]. In later work, it was demonstrated that under ideal conditions (i.e. looking at the very largest known families) that it is possible to address the problem of globular protein folding based solely on contact information derived from evolutionary information [7]. Results showed best Cα-RMSD (root-mean-square deviation of Cα atoms) of 2.7–4.8 Å on a small set of 14 globular protein targets (and one transmembrane target). This has shown the potential power of contact predictions and their importance in facilitating the survey of protein conformational space, but provides limited information on the future potential of the approach due to the very small data set.

Although the global entropy model of Weigt and colleagues was an important breakthrough in solving the indirect coupling problem in sequence-based contact prediction, more recent approaches have built on these ideas to produce even more effective contact prediction methods. In this work we used the PSICOV method to tackle indirect coupling effects. PSICOV [20] makes use of sparse inverse covariance estimation techniques (specifically the graphical Lasso procedure), which adds a powerful additional constraint to those exploited in maximum entropy methods, namely a *sparsity* constraint. It's known (and self-evident) that the true network of contacts in a protein structure is sparse, and so it is only common sense that the predicted contact map should also be sparse. However, the importance of *sparsity* constraints goes beyond a simple desire to replicate what we observe in reality. By constraining the inverse solution to be sparse, we insist that the underlying statistical model be as simple as possible, which applies the broad concept of maximum parsimony, common in many areas of evolutionary biology, to the problem of contact prediction. From a more theoretical viewpoint, there are an astronomically large number of possible complex models which can accurately reproduce the observed data (i.e. the pattern of substitutions seen in a MSA), but only a very small number of simple models have the same ability to reproduce the observed data. By avoiding over-fitting in this way, a bane of many machine learning applications, PSICOV is able to identify directly coupled co-varying columns in MSAs and thus extract contact information. It predicts contacts with an accuracy of up to 80%, regardless of contacting residues sequence separation, provided that sufficient numbers of homologous sequences to the target sequence are available (500 diverse sequences being suggested as the lowest bound).

Recent advancements in intra-protein contact predictions [6], [7], [20], [21], and important breakthroughs in the prediction of transmembrane protein structures [22], [23] prove that the approach of utilizing genomic-scale sequence information to infer residue coupling information can substantially aid researchers in building accurate 3-D models of proteins without requiring homologous templates (see the review by Marks et al. [24] for further details). In the case of transmembrane proteins, contact information combined with secondary structure predictions are sufficient to obtain correct predictions even for large (>500 residues) protein domains [22]. The amazing success in predicting transmembrane structures is clearly a result of the limited range of architectures seen in transmembrane proteins (mostly up-down helical bundles) due to the constraints of the lipid bilayer. For globular proteins, however, residue-residue contacts alone are likely to be too scarce to produce reliable results for large globular proteins [7] due to the significantly larger number of degrees of freedom enjoyed by this class of protein structure.

There have, of course, been a multitude of approaches to the *de novo* structure prediction problem. For example, such methods include ones based on all-atom representations and physical potentials (e.g. UNRES) [25], coarse-grained lattice models (e.g. CABS) [26] and, most successful thus far, fragment assembly-based methods (e.g. Rosetta and FRAGFOLD) [27], [28], [29], [30], [31]. Here, we report a comprehensive study of folding capabilities with regard to globular proteins, where we use our own implementation of fragment assembly (FRAGFOLD) as a folding engine, in attempt to investigate how to most effectively exploit information from predicted residue-residue contacts. Very briefly, contact predictions generated by PSICOV [20] are transformed and embedded into the standard set of FRAGFOLD energy terms [30], [31]. To go beyond the early observations made by Marks et al. [7], we performed our study on a diverse set of 150 monomeric globular proteins each comprising a single (and unique) Pfam domain [32]. We show that the addition of contacts substantially enriches the population of correctly predicted protein structures, both compared to the use of contacts alone, or with the original FRAGFOLD potentials alone.

Recent approaches taking advantage of global methods for predicting coevolution between residues focused predominantly on the principle, that the use of contacts enables sequence-based identification of limited protein folds. The issue of how *de novo* methods can best be generally improved by the use of inferred contact information, or how to effectively exploit the emerging data have not yet been adequately explored. Here, we attempt to address both of these issues, presenting a comprehensive study over diverse globular protein families and assessing the results in terms of how to utilize the covariance information most effectively for future blind *de novo* predictions. We also present a complete methodology enabling such predictions and the subsequent quality assessment of generated models. The current (and possible future) limitations of such approaches on a genomic scale, given limited sequence information and its growth, are also discussed.

## Results and Discussion

In this section we describe the effect of adding residue-residue contact information to aid fragment-assembly predictions of globular proteins using FRAGFOLD. We demonstrate the cases where the improvements are substantial but also try to investigate what causes the approach to fail. We also show that the quality of predictions can be assessed on multiple levels, resulting in high confidence evaluation of blind predictions. Finally, we show how best to achieve correct predictions using predicted contact information.

### The protein test set is comprehensive

In this study, a diverse set of 150 globular proteins was used as targets. The average length of a protein chain in this set is 145 residues. The set represents diverse folds, each coming a different Pfam domain family [32]. For a detailed description of the set, see Materials and Methods section.

### Folding with the addition of residue-residue contact information improves the predictions significantly

A benchmark was performed on the full 150 protein dataset. To ensure the study is representative of real blind prediction problems where no information about the test set structures is known *a priori*, duplicate or similar sequence fragments to the target dataset were removed from the standard FRAGFOLD fragment library (see Materials and Methods section for details). Simulation parameters were optimized just once prior to the start of benchmarking and not adjusted on case-by-case basis. Of course, for real problems there is no reason to avoid adjusting parameters on a case-by-case basis, but for objective benchmarking this was avoided.

**Figure 1:**
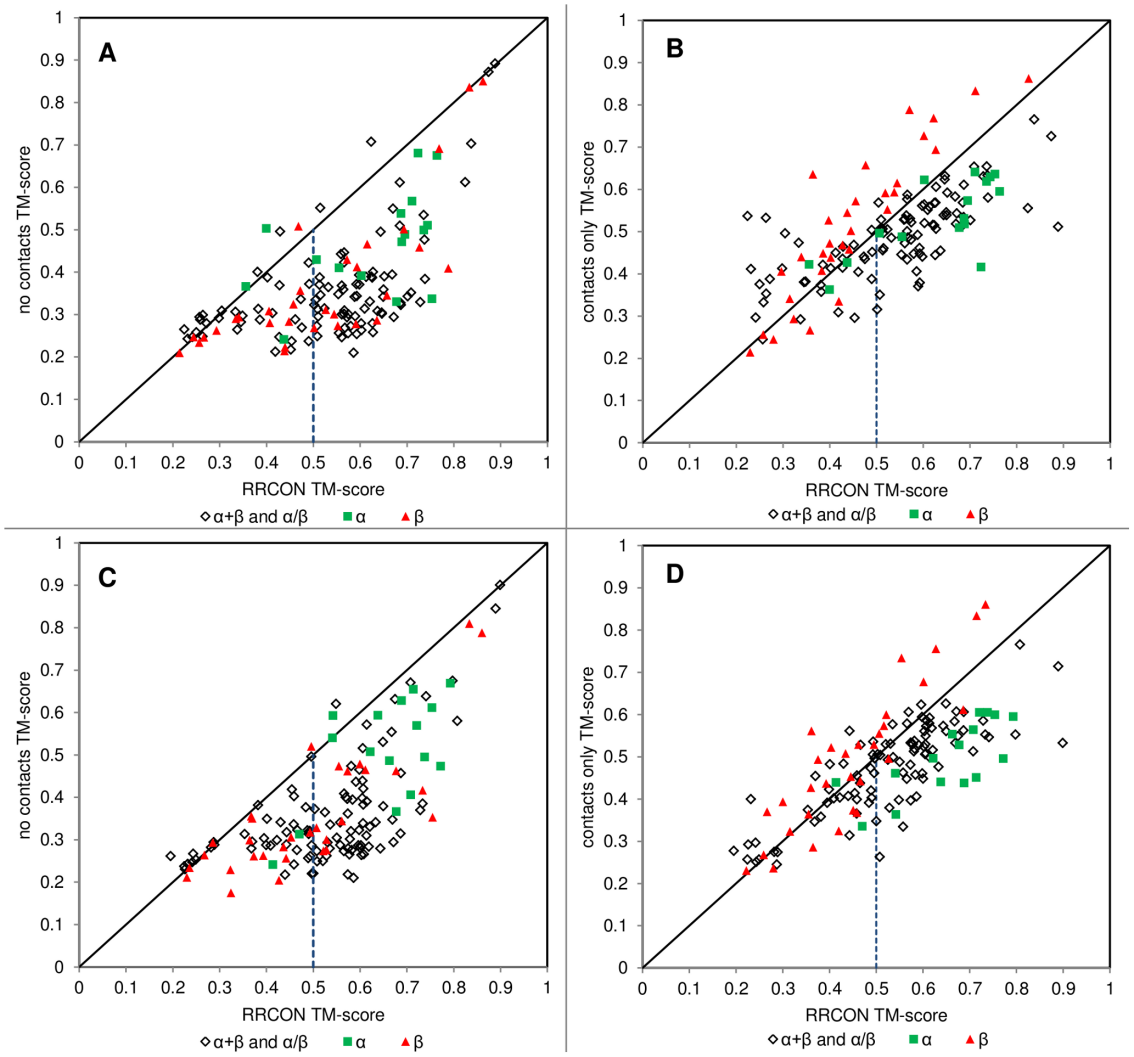
Folding with and without the use of predicted contacts. **A**. TM-scores obtained for best top-5 predictions (on the basis of calculated final energy) without (no contacts) and with residue-residue contact (RRCON) term are compared (combined *all* and *sequential* contacts; explained in the text). Three results are significantly better (TM-score difference >0.05) without the use of contacts: 1hh8A, 1m4jA, 1m8aA; upper from the diagonal. **B**. Shows contact only best top-5 TM-scores in comparison to *combined* contacts FRAGFOLD results (best top-5 energy). **C**. *Combined* RRCON results compared to no contacts results assessed on the basis of best TM score in top-5 largest clusters. **D**. *Combined* RRCON TM-score against contacts-only approach TM-score (best top-5 clusters). Diagonal lines indicate identical results. Vertical dashed lines indicate correct prediction boundary (TM-score ≥0.5). The area below the diagonal and right of the dashed line encompasses all correct predictions. Targets are grouped by fold: green squares – α-proteins, red triangles – β-proteins, diamonds – α+β and α/β proteins. Overall, 100 targets out of 150 were correctly predicted.

**Figure 2:**
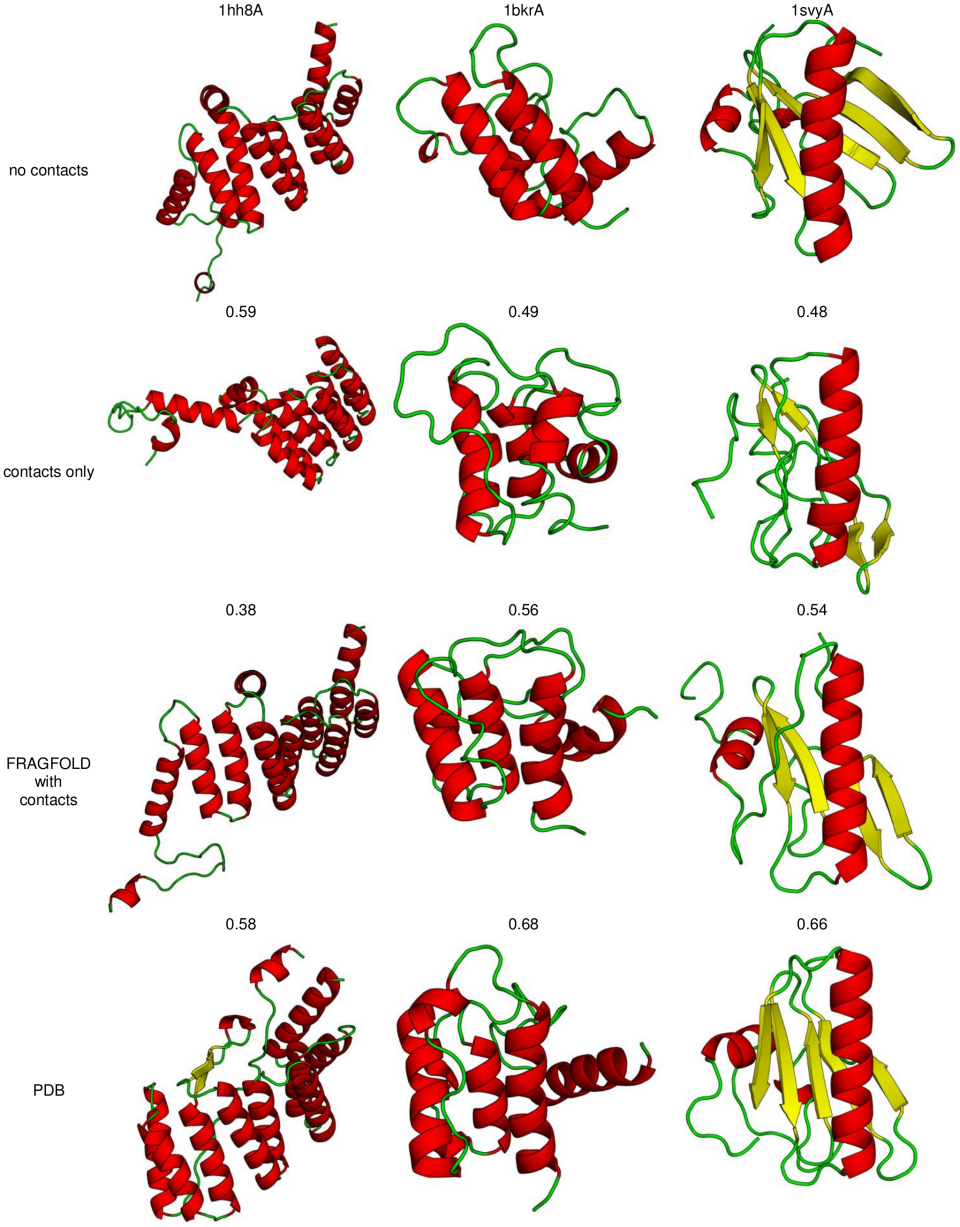
Sample results of FRAGFOLD without contacts, contacts-only methodology and both statistical and contact potentials. Below each structure its TM-score is given. 1hh8A is presented in the first column. It is a case where TM-score of no contacts structure is higher than FRAGFOLD with contacts potential (0.59 and 0.58, respectively). Targets 1bkrA (second column) and 1svyA (third) exhibit a progression of TM-score from FRAGFOLD utilizing only statistical potentials (top row), FRAGFOLD contacts-only (second row) folding and folding with both, statistical and contacts-derived potentials (third row). Such progression is expected and observed in most of cases throughout the test set.

Figure 1 shows a comparison of the best top-5 models (single highest TM-score model picked from the 5 lowest energy models in an ensemble) between FRAGFOLD with and without the predicted residue-residue contact energy term (Figure 1A and 1B). Also, a more traditional energy-independent approach was considered by clustering the ensemble of models and taking the highest TM-score model from the 5 largest clusters (best top-5 clusters; Figure 1C and 1D). Results emerging from clustering (TMclust method) proved to be inferior to those generated on the basis of calculated energy (Table 1). Because of that and due to the uniformity the energy-based approach was carried out throughout the rest of analyses and calculations. All results are assessed on the basis of TM-score [33]. Initially for FRAGFOLD without residue-residue contact prediction information, 21 out of 150 proteins had correctly predicted fold (best top-5 TM-score ≥0.5) [34]. After applying the residue-residue contact term (RRCON) there were 79 additional correct predictions, yielding 66.7% correct predictions accuracy across the whole set (Table 1 and right side trapezium in Figure 1). It is clear that RRCON improves the quality of predictions, with some cases resulting in near-perfect, or at least substantially better predictions, but also some results were not significantly better (TM-score >0.05, determined on the basis of mean standard deviation of TM-score for models generated for each protein) than without RRCON (14 cases; 11 of the cases having a statistically insignificant TM-score difference) and 3 cases where structure is better predicted without the use of contacts (1hh8A, 1m4jA, 1m8aA). The N-terminal domain of neutrophil cytosol factor 2 (1hh8A) has an alpha-horseshoe architecture, which suggests possible sampling difficulties and although the no contacts result is better in terms of TM-score (0.50 no contacts; 0.40 RRCON), both structures exhibit major features of the fold and although the target is a difficult modelling case, comparing best models generated by each method the quality difference is far less significant (Figure 2, left panel). The full set of results is included as supplementary data (Table S1). Sample predictions are presented below (Figure 2). The middle and right panels present typical results, where a progression in the quality of results is observed as contact information is introduced. Two other significant cases (1m4jA, 1m8aA) exhibit smaller variations depending on the method. The latter case (1m8aA; 0.71 no contacts; 0.62 RRCON) is clearly a sampling case and both predictions yield correctly identified fold of the protein. In case of 1m4jA (0.49 no contacts; 0.43 RRCON) the differences are modest and both close to the 0.5 TM-score boundary of this generally difficult target having 3-layer (aba) sandwich fold. Overall results generated by FRAGFOLD with RRCON yield a significant improvement over recent comparable methods utilizing residue-residue contacts (e.g. EVfold [7], Table S2).

**Table 1:** Improvements in predictions.

	best	energy		clustering	
		**best top-5**	**best top-5 ≥0.4**	**best top-5**	**best top-5 ≥0.4**
**no contacts**	24.67%	14.00%	26.67%	16.00%	29.33%
**contacts only**	63.33%	48.00%	79.33%	45.33%	74.67%
**with residue-residue contacts:**					
**all**	72.00%	58.00%	74.67%	56.67%	74.67%
**sequentially introduced**	66.67%	58.67%	78.00%	50.67%	66.00%
**combined**	78.00%	66.67%	82.67%	62.00%	82.00%

Comparison between fractions of correctly predicted models (TM-score ≥0.5 or 0.4 when noted) among best, best top-5 and best top-5≥0.4 TM-scores. Best top-5 results are analyzed as 2 groups: derived on the basis of calculated final energy (*energy*) and on the basis of cluster size (*clustering*). Results without the use of residue-residue contacts, only with the use of residue-residue contacts, or with: all predicted contacts included for the whole duration of simulation (*all*), contacts sequentially included as the simulation proceeds (*sequential*) and combined results taking advantage of both approaches are compared. Best results are predictions with highest TM-score from the whole generated ensemble, best top-5: the highest TM-score value from 5 lowest energy models (or 5 largest clusters) in an ensemble.

### Contact-only folding is less effective than when used alongside statistical potentials

Results presented above show that the addition of contact information has a great impact on the quality of predictions. To verify whether contacts alone can act as an objective folding energy function, an experiment was performed where the folding was done exclusively with the use residue-residue contact terms (ie. FRAGFOLD fragment selection and simulated annealing engine, but only RRCON having non-zero contribution; see Materials and Methods section) on the same dataset. Similar claims were presented before by Marks *et al*. [7]. Prediction accuracy in this case was 48% (Table 1). Contact-only predictions perform much better than FRAGFOLD without contacts, but the overall performance of the combined FRAGFOLD with RRCON methodology still outperforms both limited approaches. The combined approach fails in only 31 cases across the test set, and is thus clearly able to take advantage of both sources of information, coping with cases where there are insufficient constraints either from FRAGFOLD itself or predicted contacts alone. Comparing contact-only results to FRAGFOLD without RRCON, the improvements are more modest (Figure 1B, Figure 2), although overall there are some cases where the contacts only approach showed better results. It is clear that combination of the FRAGFOLD energy terms with additional RRCON terms yields significantly better results (also in comparison to similar methods utilizing contact predictions; Table S2). Still, there are proteins where the contact-only approach produces better models – addressing this problem, without changing parameters on by case basis, can be done by sequential introduction of contacts rather than relying on contact-only predictions which do not have indicators as to when apply this methodology. We discuss this later in this section.

### Optimal usage of contact information with FRAGFOLD

**Figure 3:**
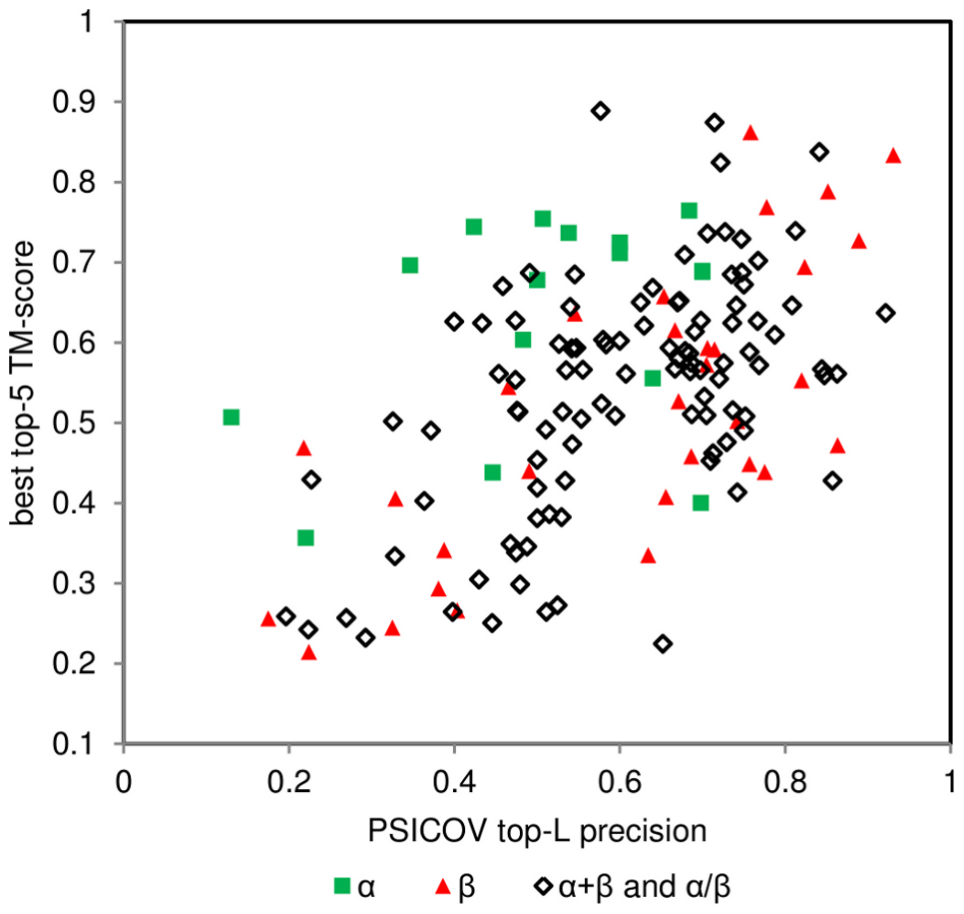
By fold comparison of best top-5 TM-score with PSICOV top-L precision. Red triangles – β proteins, green squares – α proteins, diamonds – α+β proteins and α/β proteins.

The presented results reveal a high degree of improvement in *de novo* protein structure prediction due to the usage of predicted residue-residue contacts. However, an important question to ask is what is the best way of combining predicted contacts with the FRAGFOLD objective function. In trying to address this before carrying out the benchmark, we tried a large number of different approaches on a limited subset of cases, as described in the Materials and Methods section. Of course it is impossible to know whether we have found the absolute best design for a hybrid scoring function, but we can say that the eventual choice was the best out of very many combinations that we tried. These combinations centred around 3 main choices: i) Should all predicted contacts be used or only the most confidently predicted ones? ii) How heavily should contact information be weighted in comparison with the standard FRAGFOLD energy terms? iii) What function should be used to transform predicted contacts along with their associated precision estimates into good pseudo-energy terms? After trying various combinations, we found the optimum performance on the small validation set to be as follows:

1. Use the full list of predicted contacts produced by PSICOV, as any of the contacts can potentially contribute to the determination of a correct fold. Artificially truncating the list is likely to remove useful information. The weight of a given predicted contact is determined by positive predictive value (PPV) which then serves as an argument for the contact energy terms. Similar observations also concern limiting the list of contacts to ones of a given range (e.g. only short-range or long-range contacts). Although, as we present below, long-range contacts are crucial and highly informative on their own, limiting the contact information to them significantly impairs the predictions (data not shown).
2. The relative weight of RRCON should be equal to the total of all other FRAGFOLD terms. In practice, FRAGFOLD adjusts weighting parameters on the basis of ratios of standard deviation of every potential term with relation to short-range pair-wise potential component [30]. Physical potential terms contribute equally to the final structure, as evolutionarily-derived residue contact information. As contact information comes with varying quality (characterized by PSICOV precision; Figure 3) RRCON contribution can be either under- or over-fitted, as contact-only folding results demonstrated. The best solution in the general case thus seems to be equalising contact and physical term weighting. It is quite possible that some further improvement could be obtained by varying the weighting of contact terms depending on the estimated accuracy of the contact list, but none of the simple approaches we tried were successful.
3. The contact energy term should be scaled according to the estimated precision of the contact and should also penalize unsatisfied contacts. Our final selected pseudo-energy term is given in the Materials and Methods section.

### The contacts utilized in this method are fitted optimally

**Figure 4:**
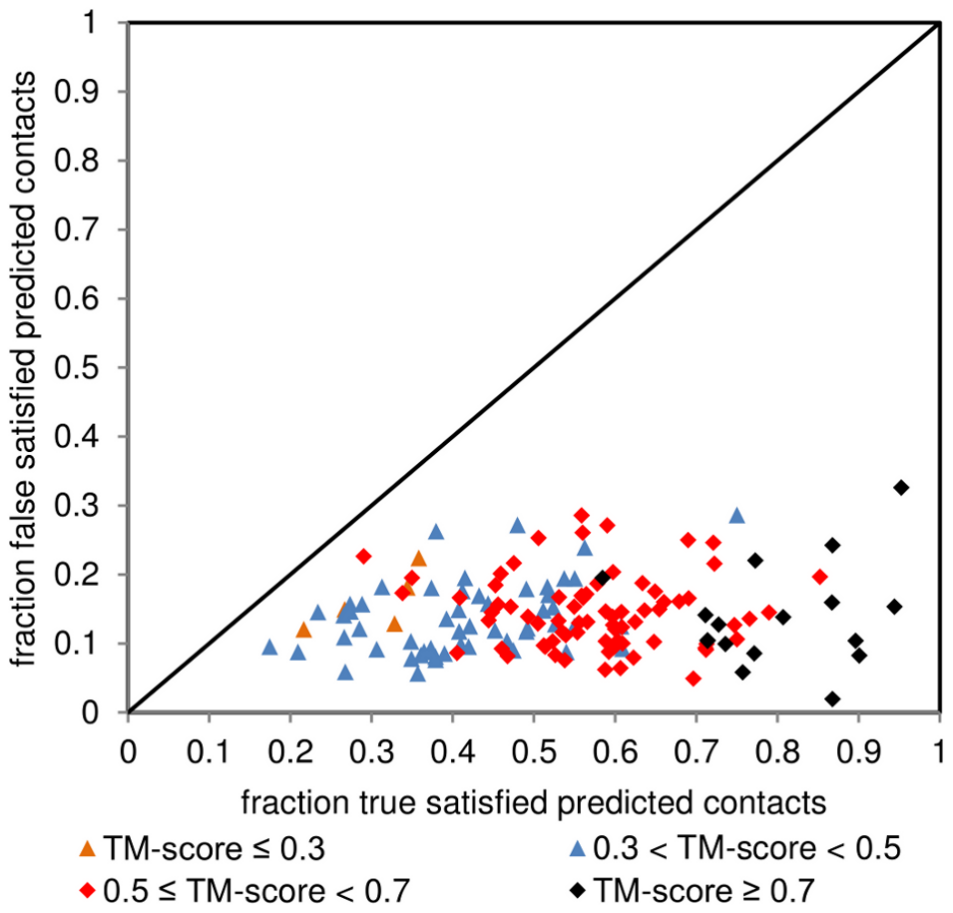
Scatter plot of top-L true against top-L false satisfied predicted contacts. Equal numbers of contacts in each group (true or false) per protein are compared against each other. The diagonal line indicates equal contribution boundary. Orange and blue triangles represent incorrectly predicted targets (TM-score ≤0.3 and 0.3<TM-score<0.5, respectively), red and black diamonds correspond to correctly predicted targets (0.7>TM-score≥0.5 and TM-score≥0.7, respectively).

In the dataset there are no cases where more than a third of top-L (from the list of contacts sorted by descending PPV value; L is the length of the protein) predicted false contacts would be satisfied (Figure 4). At the same time, amount of true top-L predicted satisfied contacts reflects well the quality of models. Best predictions (TM-score ≥0.7) satisfy at least 70% of true predicted contacts in all cases. In this regard the contacts are not over-fitted (there are no cases with more false than true predicted contacts satisfied), although we acknowledge the notion that any satisfaction of false contacts can be considered over-fitting.

The converse hypothesis has also to be tested – are the contacts under-fitted? If that was the case, the increase of residue-residue contact weighting in relation to other potential terms would not improve the model quality. A boundary condition here is contact-only folding, which was already discussed and indeed there are cases where contact contributions are under-fitted (Figure 1B). This scenario however is not very common (30 cases in total generating difference in TM-score greater than 0.01, including cases where both approaches produce models with TM-score ≥0.5), and it is safe to state that in general terms contacts are well fitted into the method. By-case adjustments are likely to fix this issue or, as we present in the next sub-section, an alternative approach to introduce RRCON by including contact information sequentially.

### Sequentially introduced contacts improve predictions in some cases

Two approaches to introduce predicted contacts were used: in the first, the full set of predicted contacts was added into the FRAGFOLD energy function (later referred to as *all* contacts) and in the second, the contacts were introduced sequentially. In the *sequential* case, only short range contacts are considered at the start of the run, with the range of considered contacts being increased linearly with each cycle. Results from both approaches can be analysed together i.e. where best top-5 results are selected from the combined population of results, which we refer to as *combined*.

**Figure 5:**
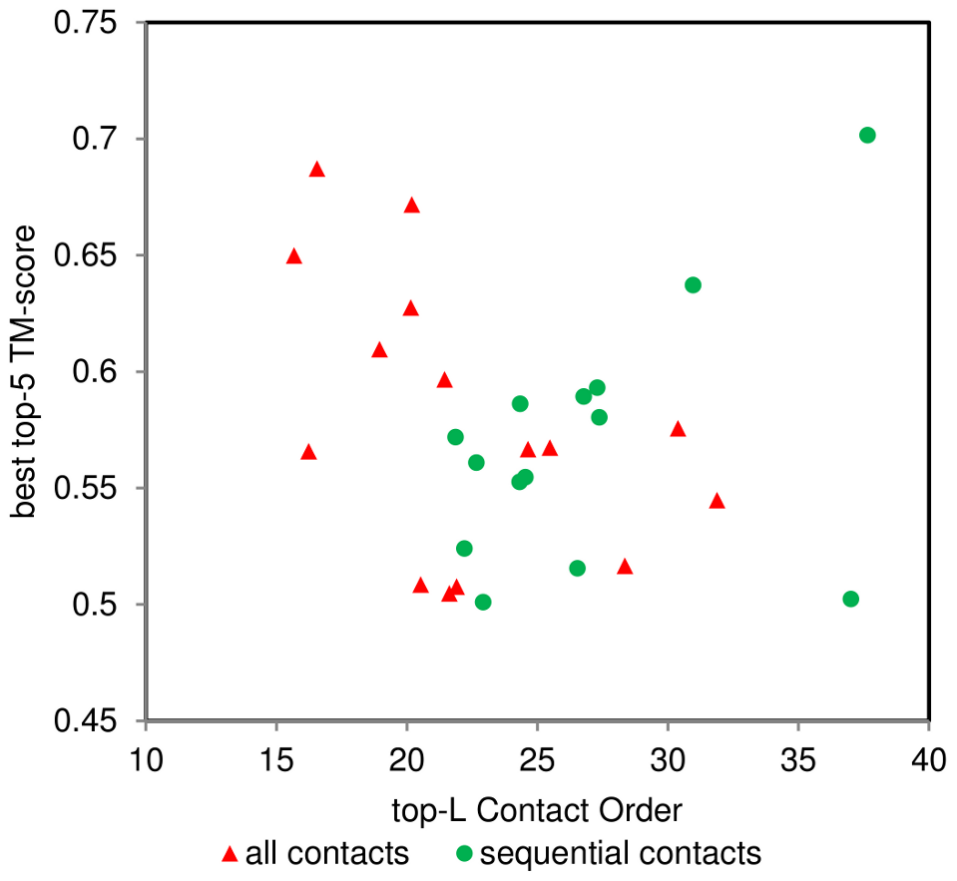
Top-L contact order compared against TM-score for all contacts and sequential contacts targets. Contact order calculated across the whole chain length and reflects the relative contribution of long-range contacts (predicted) in the whole structure. Cases where all contacts produce correct topology but not sequentially introduced (all contacts; blue diamonds), and where only sequentially introduced contacts produce correct topology (sequential contacts; red squares) are compared. It may be observed that the former case exhibits better results for low (<25 top-L CO) contact orders, while the latter for higher contacts orders (approx. 25 top-L CO and more).

Table 1 shows the results of a*ll* contacts and *sequential* contacts calculations. Nevertheless, basing on energy or clustering it is possible to pick a better model generated by these two alternative approaches, hence improving overall performance (Table 1). This however requires twice as many computations and is not always feasible. Contact order (CO) might help to choose the correct approach (Figure 5). CO is defined as an average separation between contacting residues divided by the length of a protein [37]. The greater CO is the more long-range contacts are present within a protein. Here, predicted contact order is studied, calculated on the basis of top-L predicted contacts (L being the length of the protein). An overall trend is observed here – low top-L contact orders (top-L CO<20) tends to favour *all* contacts approach, while high top-L contact order (top-L CO>25) favours *sequential* contacts rather than *all* contacts approach. There is no clear rationale for this, however some hypotheses might be presented. When there are more long-range contacts than short and mid-range ones (high CO), introducing all contacts at once disrupts satisfaction of shorter contacts in favour of long-range contacts. Sequential formation of contacts enables a protein to obtain a correct fold reducing the noise from long-range contacts.

Also, as previously mentioned, *sequential* contacts enable the method to overcome under-fitting problems – contacts introduced as simulation progresses can be satisfied to a larger extent (i.e. better fitted), as opposed to *all* contacts approach – and improve results without altering weighting parameters or the folding framework in general. Changing parameters or the overall framework is not suitable for blind predictions where the quality of predicted contact information cannot be readily assessed, or in large-scale folding experiments.

### Incorrect predictions can be classified as sampling or contact-related problems

**Figure 6:**
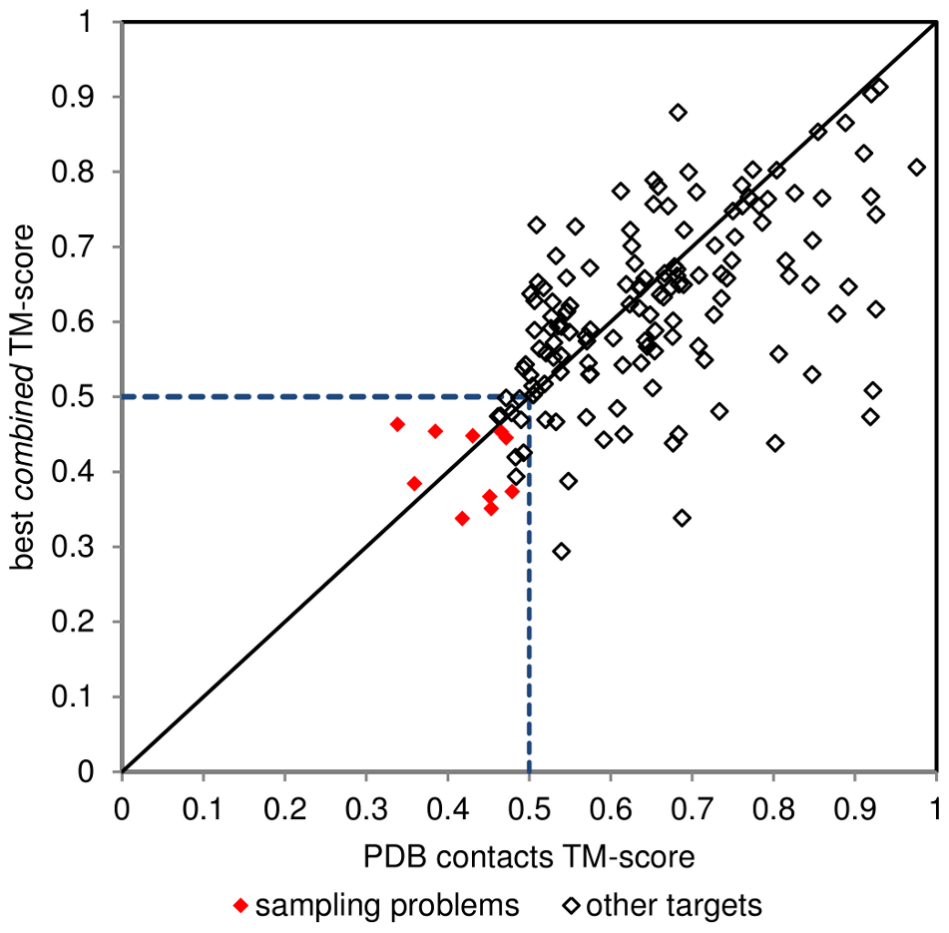
TM-scores of best results obtained using predicted contacts compared with folding results aided by contacts extracted from PDB structures. Red diamonds indicate identified sampling problems. Contacts extracted from experimentally solved structures (PDB contacts) clearly improve the predictions (points below the diagonal).

There is no single explanation why some cases do not improve (or get worse) when we add contact information to statistical potentials. Generally, unsuccessful predictions might be attributed either to sampling, or contact-related problems. This was tested by substituting predicted contacts by contacts extracted from crystal structures. Cases where the use of structure-derived contacts rectified unsuccessful predictions based on predicted contacts can be classified as contact-related problems (Figure 6). If the real contacts do not improve a prediction sufficiently to obtain a correct fold, then we assume the failure is due to a lack of conformational sampling. Overall, half of classified problems are contact-related (11 cases), while the other half are sampling difficulties. Table 2 compares the cases where these types of problems were identified.

**Table 2:** Sampling and contact-related problems.

protein	no contacts TM-score	RRCON TM-score	fold*	top-L PSICOV precision	fold architecture
**sampling problems** #					
1aoeA	0.30	0.45	α/β	0.50	3-layer sandwich
1d4oA	0.34	0.44	α/β	0.40	3-layer sandwich
1dixA	0.34	0.34	α+β	0.27	alpha-beta complex
1fl0A	0.36	0.37	β	0.63	beta barrel; OB-fold
1gzcA	0.31	0.38	β	0.40	jelly roll sandwich
1hfcA	0.29	0.45	α+β	0.47	3-layer sandwich
1i1jA	0.29	0.46	β	0.49	roll (barrel)
1jbkA	0.32	0.37	α/β	0.22	3-layer sandwich
1kqrA	0.28	0.35	β	0.18	jelly roll sandwich
1rybA	0.33	0.45	α/β	0.74	3-layer sandwich
3dqgA	0.31	0.47	β	0.76	sandwich
**contact-related problems** ##					
1behA	0.28	0.44	β 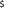	0.45	alpha-beta complex
1c52A	0.33	0.44	α	0.45	orthogonal bundle
1dqgA	0.26	0.29	β	0.22	trefoil
1ej8A	0.38	0.46	β	0.33	immunoglobin-like sandwich
1fcyA	0.37	0.47	α	0.22	orthogonal bundle
1hxnA	0.35	0.45	β	0.38	4 propellor
1i71A	0.28	0.42	β	0.49	beta barrel (disulfide rich)
1jyhA	0.38	0.39	α+β	0.47	alpha-beta barrel
1whiA	0.34	0.45	β	0.65	beta barrel
2arcA	0.30	0.34	β	0.33	jelly roll sandwich
2phyA	0.40	0.44	α+β	0.50	2-layer sandwich

Only cases where clear allocation to one of these two cases can be made are shown.

*obtained from SCOP database [35] and verified in CATH [36].

# supplying real contacts extracted from PDB does not ensure a correct prediction.

## PDB contacts enable correct prediction; the shortage or incorrectness of contact information results in poor prediction.

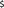
SCOP: β, CATH: α+β.

Sampling problems may be attributed to the topological complexity of some targets. These include Ig-like or beta barrel folds and sandwich architectures. In the case of the sandwich fold the two anti-parallel beta-sheets in the protein have flexible links, whereby small variations in torsional angles cause significant changes in the orientations of the two sheets. To further verify whether the cases identified as sampling problems are indeed due to sampling, not fragment availability (see Materials and Methods sub-section Folding for methodological details) we performed a verification of the quality of fragments on the 150 protein set. Best fitting fragments from both supersecondary and fixed-length fragment sets of Fragfold library were fitted onto the PDB (experimental) structures and a mean RMS Distance Matrix Error (DME) value for each protein and each set (supersecondary and fixed-length) was calculated. Then, we constructed a list of descending RMS DME values for each type of fragments (Table S3) and also identified outliers (points 1.5 times or more the interquartile range above the third quartile, where the interquartile range is the difference between the third and first quartile) in each class. Fixed-length fragments showed no outliers, while supersecondary fragments produced 5 (Table S3). Among the identified outliers 2 cases were attributed to contact-related problems (Table 2 and Table S3) and 1 case was a sampling problem (1i1jA). Overall, 9 out of 11 identified sampling problem cases (and 8 out of 11 contact-related) are within the top 50% of both rankings (supersecondary and fixed-length fragments; Table S3). Clearly, sampling problem cases are more populated amongst fragments not fitting closely onto their corresponding experimental structures, but this alone is not enough to attribute poor Fragfold performance to limited fragment availability. Fragfold also takes advantage of small fragments and they could supplement well regions where larger supersecondary or fixed-length fragments do not generate an optimal conformation. The identified sampling problem outlier (1i1jA) – melanoma inhibitory activity protein – exhibits a large fraction of loops (62%) and this is the likely cause why this protein was found to have a poor fit against Fragfold fragment library. The intrinsic drawback of any coarse-graining (here, the use of fragments) is the trade-off between computational efficiency and resolution such method could achieve.

The second group, contact-related problems, can be easily linked with low PSICOV contact prediction precision. All but one such cases have 0.50 or lower top-L PSICOV contact prediction precision. The outlier (1whiA) is a 122 residues long beta barrel protein that has a relatively high top-L PSICOV precision of 0.65. The likely cause of this target being identified as a contact-related problem is the presence of a 9 residue long loop connecting 2 β-sheets which is not constrained by any contacts. It should be noted, that although a poor prediction due to a contact-related problem is very likely to be caused by a low PSICOV contact prediction precision, not all low PSICOV precision cases produce low TM-score models. A good example could be 1fk5A (nonspecific lipid-transfer protein) where regardless of a very low PSICOV precision of 0.13, FRAGFOLD statistical potentials are sufficient to produce a good prediction (TM-score  = 0.51).

### Results correlate with contact prediction precision

It has already been shown that the number of sequences in an MSA correlates moderately with PSICOV precision [20]. This has a clear justification – the more sequences in the MSA, the richer the evolutionary information will be and hence the more accurate the calculated covariance matrix will be. We should also expect that as the contact prediction precision gets higher, the better the fold predictions should be (aside from those targets suffering from sampling problems).

Indeed, when looking at the prediction quality dependence on the PSICOV top-L contact precision a modest correlation exists (Spearman's ρ = 0.48) (Figure 3). This improves if individual folds (α, β, α/β and α+β) were analysed separately. The highest correlation (Spearman's ρ = 0.72) may be observed for β proteins. This may be rationalized by the fact, that these proteins are generally difficult modelling targets and so the baseline model quality (i.e. without contacts) is much lower, and so the improvement from contacts is likely to have greatest relative impact. Conversely, α proteins, generally easier modelling targets, do not benefit greatly from additional RRCON information, as secondary structure predictions and correct fragment selection are the major factors in folding of these proteins. A further explanation is that the long range contacts in β sheets will generally be shorter and with lower variance than those between helices due to the extra geometric constraints afforded by the hydrogen bond network. However, opposing this second explanation is the fact that the sheets themselves can change in terms of gross shape between distant homologues, thus lowering the observed model quality score. This simplistic approach has several pitfalls. During the folding process not only predicted contacts contribute to the final structure, although they play an important role as it was shown before. Contact information is not needed in all cases to find a correct fold (leftmost green square in Figure 3), or in some cases it is not sufficient to produce a correct prediction (sampling problems; see subsection “Incorrect predictions can be classified as sampling or contact-related problems”). For outliers in Figure 3 not imposing sampling problems it can be speculated that although contacts occupy the top-L range they are not predicted with large enough confidence (PPV) to impact the structures enough. The same would apply to cases where high PPV or highly populated predicted contacts occupy a single niche (e.g. 1aapA having top-L PSICOV precision of 0.86 and best top-5 TM-score  = 0.43 has a high CO = 36.4).

It would be more desirable to observe a dependence of the size of a MSA on the quality of predictions (Figure S1). Unfortunately, the correlation in that case is weak (ρ = 0.19) and is of little use for any predictions about the possible quality of generated models. It is due to the fact that the size of a MSA moderately correlates with the quality of contact predictions (PSICOV precision) and the precision of contact predictions, in turn, correlates with the quality of models. This kind of complex correlation between MSA and the quality of models results in poor dependency that is observed for these variables.

### Long-range contacts have the greatest impact on the quality of models

**Figure 7:**
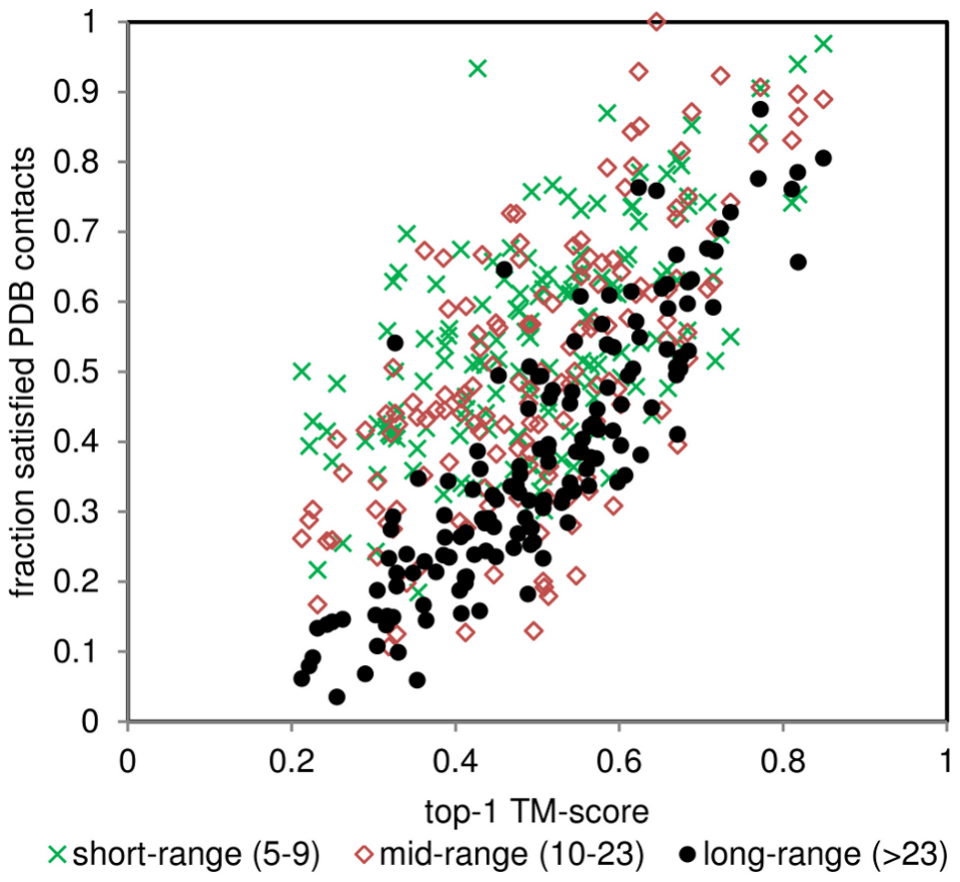
Post analysis of contact satisfaction. Contacts divided into 3 groups (short, mid and long range contacts) show dependency of the final (top-1; lowest energy in an ensemble) model on the fraction of satisfied real contacts (extracted from reference PDB files).

Issues mentioned above can be better assessed if contacts of different ranges are analysed separately. Posterior contact satisfaction analysis (the contacts present in a model, that exist in the native structure) shows that all contacts relate well with the quality of model regardless of the contact range (short-range contacts – 5–9 residues apart; mid-range contacts – 10–23 residues; long-range contacts – 23 residues and more) (Figure 7). Clearly, the long-range contacts show the greatest dependency with the final model quality, while short and mid-range contacts exhibit a more scattered pattern. This could be expected – long-range contacts constrain the overall fold of the protein, while shorter contacts relate to local similarities, which could not necessarily be properly arranged in space causing unsatisfactory overall prediction. It can be noted, that although long-range contacts give best estimation of the model quality the calculated correlation (ρ = 0.87 for long-range contacts; ρ = 0.50 for short-range and ρ = 0.57 for mid-range contacts) is still imperfect (Figure 7, black dots). This is only to be expected, as even correctly predicted contacts only provide loose constraints on the actual observed distances.

### The method is suitable for automated predictions

Targets with ≥0.8 top-L PSICOV precision (or generally, high contact prediction accuracy) are likely to produce models with correct topology, as long as no sampling problems are encountered (Figure 3). This, however, is unknown *a priori* for truly “blind” predictions, and so even if we cannot disentangle sampling and contact-related problems in real world prediction cases, as long as we have strong guidelines on how to identify correct models, contact-assisted prediction can still be extremely useful.

Considering top-L PSICOV (predicted) contacts, similar trends could be observed as in the case of post-analysis on extracted PDB contacts (in correlations terms short-range ρ = 0.19; mid-range ρ = 0.41; long-range ρ = 0.79). Short-range contacts have a weak impact on the quality of final model, mid-range have a stronger effect, whilst long-range contacts play a crucial role. Carrying out observations at top-L/2, top-L/5, etc. contacts distorts the overall picture, as for some targets different classes of contacts are underrepresented (i.e. cases where there are no short or mid-range contacts in top-L/2 are frequent). This good correlation between the number of satisfied long-range contacts and the quality of obtained models again emphasises the importance of long distance interactions on maintaining the fold of a protein.

**Figure 8:**
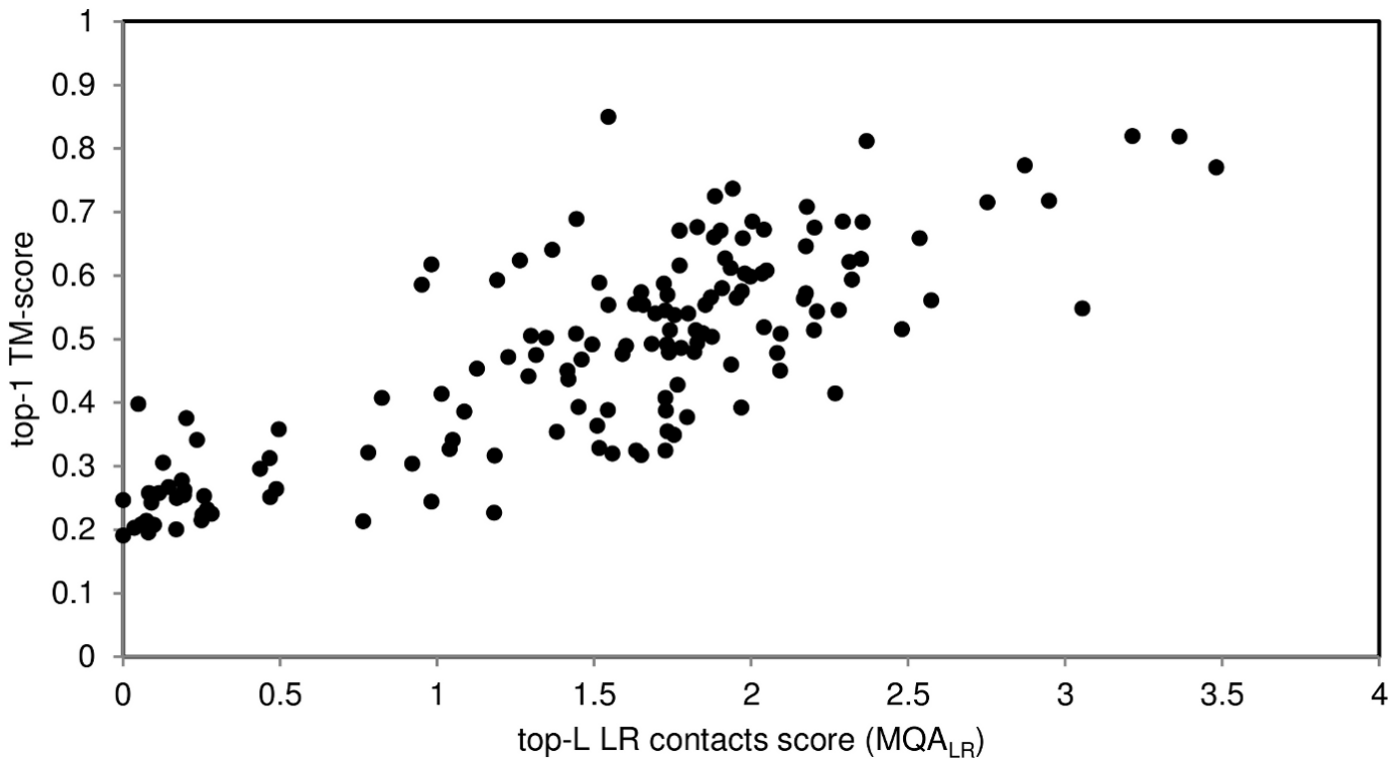
TM-score of the final (lowest energy) model against top-L long-range contact score. The score is derived basing on the length of a protein, total number of predicted contacts and the fraction of satisfied predicted long-range (>23 residues) contacts. The Spearman correlation coefficient (ρ) is 0.77.

Because long-range contacts are equally well-predicted as shorter ones [20] and they have a greater impact on the final structures generated, long-range contacts serve well as an indicator of prediction quality. In order to maximize the information content of such approach, it is useful to construct a scoring function which takes into account the total number of predicted contacts (*N_LR_*) along with the fraction of satisfied predicted contacts (*CON_LR_*).
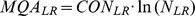
This simple quality assessment function (*MQA_LR_*) was selected from a set of tested monotonic functions as giving the best results for model assessment. *MQA_LR_* may serve as a good guideline for discriminating correct and incorrect predictions (Figure 8). Precision and recall of correct predictions (TM-score ≥0.5) of 85% can be reached with this method. Minimizing the amount of false positives by varying a threshold (but reducing the true positives fraction at the same time) gives up to 92% recall (67 true positives and 15 false positives).

**Figure 9:**
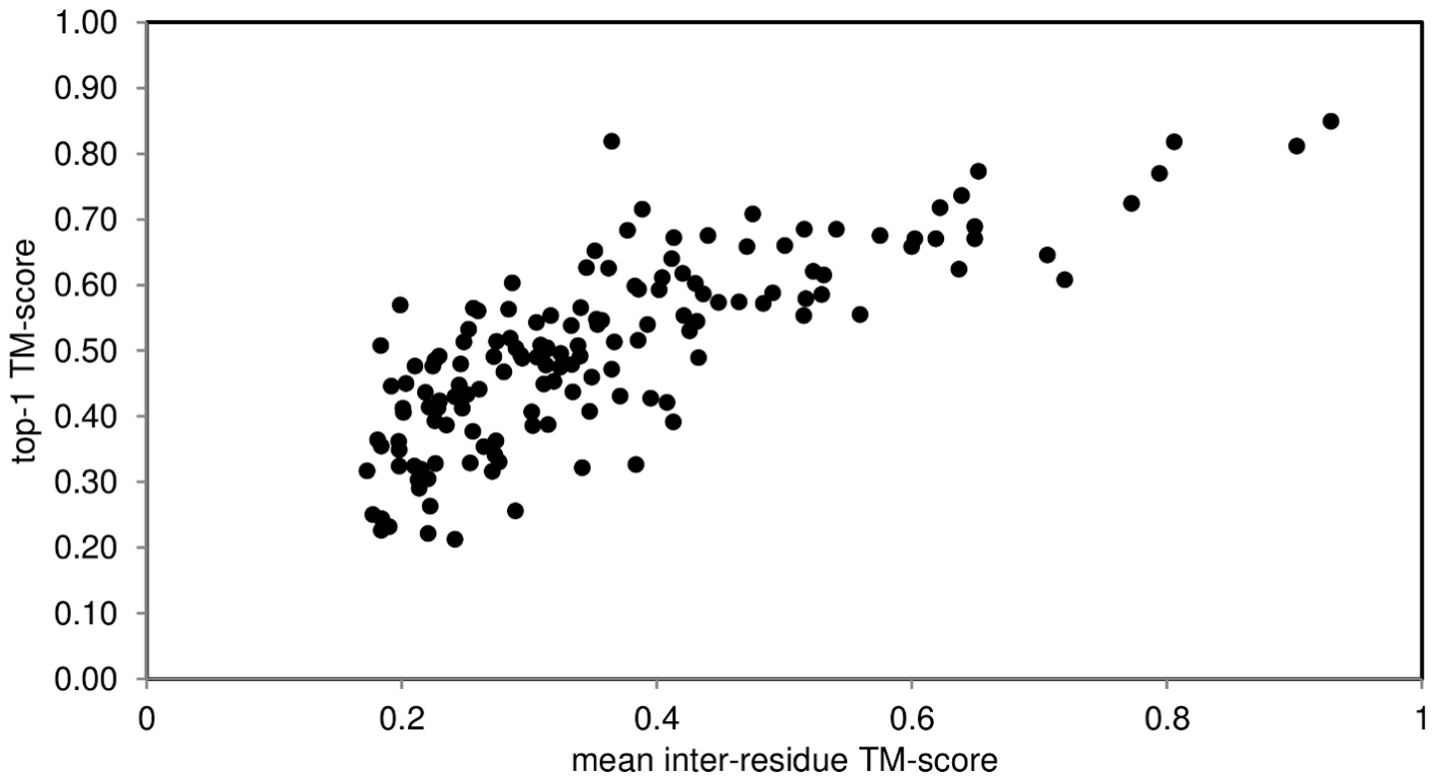
TM-score of the final (lowest energy) model against mean pair-wise TM-score within the model's ensemble. Good correlation (Spearman's ρ = 0.73) emerges from the results. Inter-residue TM-score >0.26 is likely to produce a model with TM-score >0.5.

Regardless of sampling problems, either the long-range satisfied predicted contacts score (discussed above) or mean inter-residue TM-score variations reflect the prediction quality well (mean inter-residue TM-score was calculated for each pair of models in the ensemble; as described in [22]). The plot of mean TM-scores across pairs of ensemble models and the final (lowest calculated energy) model shows a strong correlation (Spearman's ρ = 0.73) (Figure 9). Should the mean pair-wise TM-score cut-off be >0.25 the expected TM-score of the final model is ≥0.5 at 84% precision (probability of a correct model) and 58% recall (42 true positives and 8 false positives).

**Figure 10:**
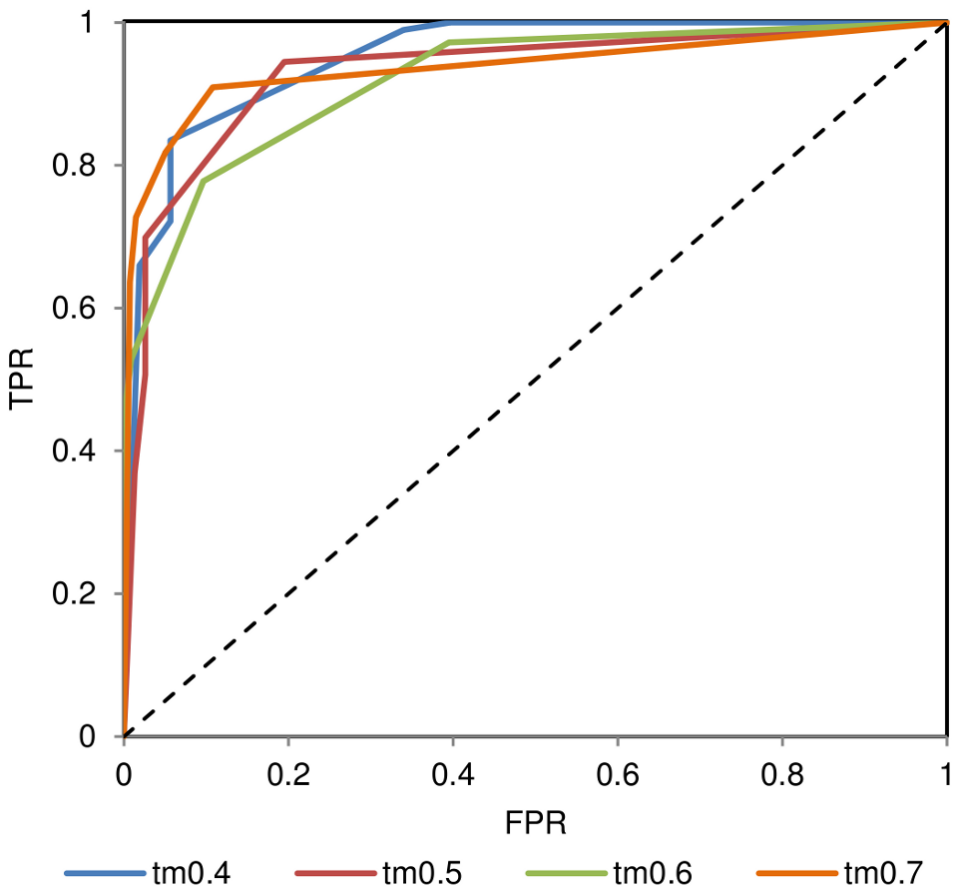
Accuracy of predictions basing on the total inter-residue TM-score and long-range contact score. ROC curves are plotted at different TM-score cut-offs. TPR – true positive rate, FPR – false positive rate. Diagonal dashed line indicates random prediction boundary.

A combined model quality assessment function (*CS*) may be constructed on the basis of two previously described functions: mean inter-residue TM-score (*TM*) and top-L long-range contact satisfaction score (*MQA_LR_*) (Table 3, Figure 10).

**Table 3:** Model quality assessment on the basis of a combined score (*CS*) derived from long-range contact satisfaction score and mean inter-residues TM-score in an ensemble of models.

TM-score	*CS*	precision	recall
≥0.4	>3.0	0.96	0.83
≥0.5	>3.4	0.93	0.77
≥0.7	>6.0	1.0	0.64

Data concerns the full 150 protein dataset.



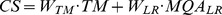
The combined model quality assessment function weights (*W_TM_*, *W_LR_*) were optimized using a genetic algorithm to maximize the precision and recall values. *W_TM_* = 7 and *W_LR_* = 1 were found to be the optimal weights for the function. *CS* exhibits higher correlation with TM-score of the final model than the two former functions alone (ρ = 0.91) and can serve as a guideline concerning assessment of blind prediction models with very high precision and recall values (Table 3).

### The role of contact-assisted predictions is likely to increase in future

In terms of future usage, we should put the presented data in a genome-wide context. Since the test set was constructed basing on Pfam, the most obvious reference point is that database. The Pfam database is a good reflection of the currently available sequence space, covering over 77% of it. The annotated portion of Pfam (PFAM-A) currently consists of 13,672 families [32]. Only about 42% of these families have at least a single corresponding PDB structure. Although Pfam covers all types of proteins, transmembrane proteins contribute to a negligible fraction of about 3% of all Pfam families (by cross-referencing to UniProt), whereas they constitute about 30% of a typical genome [38].

**Figure 11:**
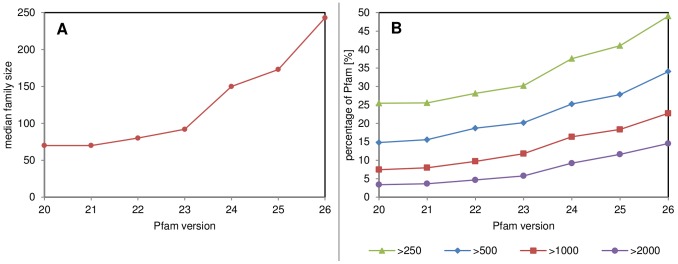
Growth of Pfam holdings from version 20. **A**. plot of the increase of median family size and **B**. percentage of Pfam with families of size above sequence length thresholds: 250, 500, 1000 and 2000 residues. In all cases an exponential growth may be observed. Currently (version 26) median family size is 248 and 34% of families hold more than 500 sequences.

**Figure 12:**
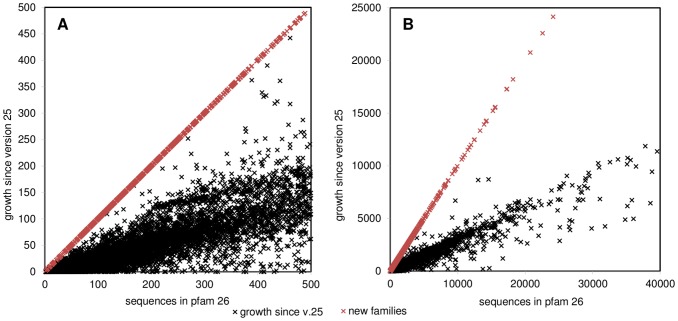
Number of sequences in Pfam version 26 in comparison to the growth since version 25. Upper line (red) indicates emerging new families not present in version 25, lower points (black) indicate a stable growth of the families in size. Not all data is shown. **A**. Region of up to 500 sequences, below the capabilities of most contact prediction methods. **B**. Region up to 40,000 sequences. Some families decrease their size (negative value on the ordinate axis), what might be attributed to redefinition of some families. Number of sequences range up to over 288,000 sequences (COX1 cytochrome c oxidase family), but with low density.

The Pfam database is growing at a high pace (Figure 11). This concerns all families, both large well-characterized ones, and small emerging with little sequences. In fact, when observing the growth of Pfam 2 linear trends may be observed (Figure 12). By sequencing and following redefinition of families the new families emerge in considerable amounts, up to around 8,000 sequences. This trend is reflected by the change of the current median family size, with 250 residues (Figure 11). At present, 34% families from the most recent Pfam release contain at least 500 sequences. Given the current growth rate by an exponential extrapolation, it can be expected to reach 50% of families will contain >500 sequences by 2015. The 500-sequence threshold is somehow arbitrary and considering no direct correlations between number of sequences and quality of models generated, as well as advances in the field of contact predictions, it cannot be treated as an absolute determinant. However previous study by Jones *et al*. shows, that when reaching 500 sequences in a MSA the precision of contact predictions drops very low [20], justifying treating 500 sequences in a family as a good guideline for the use of predicted contacts.

## Conclusions

We have explored and determined the optimal usage of predicted residue-residue contact information in *de novo* protein structure prediction on a comprehensive benchmark of 150 globular proteins. For this purpose FRAGFOLD, a fragment assembly method, was enhanced to use predicted contacts as an additional pseudo-energy term alongside statistical potentials. FRAGFOLD utilizes the predicted intra-protein residue-residue contact prediction information generated using sparse inverse covariance estimation on multiple sequence alignments, as implemented in PSICOV. Added information emerging from predicted contacts greatly enhances the predictive capabilities of FRAGFOLD, showing significant improvements over recent methods. The presented version of FRAGFOLD is able to correctly predict almost 80% of proteins in the test set (best TM-score ≥0.5 threshold) with a mean TM-score of 0.54.

Using the method described, it is possible to reliably estimate *a priori* the quality of obtained models, on a basis of the fraction of satisfied long-range contacts, correlation with mean inter-residue TM-score, or on the basis of the developed combined model quality assessment score (*CS*).

Thanks to the effectiveness and reliability of *CS* the current method is well-suited for automated *de novo* predictions enabling selection of correctly predicted folds (TM-score ≥0.5) with 93% precision and 77% recall. This is of particular importance in all practical applications, where the assessment of the quality of obtained models is indispensible. In PSICOV long-range contacts appear to be equally well predicted as short-range contacts. It helps to take advantage of the importance of long-range contacts on the fold of a protein. We showed that these long-range interactions (>23 residues apart in the sequence) play a crucial role in obtaining a correct fold and basing solely on them it is also possible to estimate the correctness of obtained models. Current study showed that RRCON term plays an equal role as all of the statistical potentials embedded in the folding engine of FRAGFOLD. Improvements in predictions coming from the utilization of both types of potentials clearly outperform any approach used alone (14% correct best top-5 predictions without RRCON, 48% with contacts only and 66.67% correct predictions combining contact and statistical potentials). Part of this increase in the amount of correct predictions was due to parallel use of alternative approaches to introduce contact information as simulation proceeds – either *all* contacts from the start, or *sequentially* from short-range to long-range contacts as simulation proceeds (i.e. from 58% using *all* contacts, to 66.67% using *combined* approach).

Considering the perspectives of contact-assisted predictions the increase in the number of available sequences alone will not solve the protein structure prediction problem. As we presented in this paper, a fraction of poor predictions emerges from sampling problems, which cannot be avoided by the improvement of the quality of contact information. These cases constitute half of the unsuccessful predictions. There is a need for better sampling algorithms, which would bypass topological problems often present in structures containing β-sheets, e.g. sandwich architectures (analysis of folds reveals that β-proteins still remain the most challenging targets, although most significant improvements due to the introduction of contacts also occur in this class).

The other conclusion concerning future blind prediction usage is: contact-related problems may be bypassed by the increase of sequence information, but there is still a need for improvement in the contact prediction algorithms as well. As it was already pointed [20], some statistical problems still are to be dealt in contact predictions. One of them being weighting of the sequences in large MSAs, as it is a computationally expensive step which is limiting the size of proteins that can be predicted with these methods. Also, better alignment algorithms would benefit the overall accuracy of contact predictions. A hope to reduce the rate of false positives in contact predictions might come from identifying signal generated by protein-protein interactions, or interactions with ligands or cofactors.

Nevertheless, uniform growth of protein sequence families collected in Pfam gives prospect for a constant increase in the predictive capabilities of this and similar methods. This is of particular importance, since the ever-increasing gap between the sequence and structure spaces [39]. It can be speculated on the basis of obtained results, that the lower bound for current predictive capabilities (since PFAM-A collects only annotated families) is in the range of 23% of current sequence space. This is based on a simple estimation – there are 34% of Pfam families with more than 500 sequences, our method is able to correctly predict approx. 67% of cases (considering best top-5 results). These speculations do not consider homology, multimeric proteins, etc. but are intended to provide an outlook of what should be within this method's reach.

Results presented here are promising and show how to use contact predictions to predict protein structures effectively. The main bottleneck remains the reliance of covariance-based contact prediction on the size of the available MSA. Although improvements in this direction have been reported, the accuracy of contact prediction for small MSAs is still far too low to be useful [40]. The study here has focussed on the modelling of single protein domains, but there is clearly a major challenge to address in the modelling of large multidomain complexes, where improved contact prediction is also likely to be beneficial. Here the bottleneck will be correct handling of orthologous relationships within large superfamilies i.e. dealing with the problem of repeats of domains appearing in different contexts. Finally, there is still a lot of room for the development of better conformational search algorithms. Even fragment-assembly methods are still relatively inefficient when faced with large complex topologies. In these cases, it may be better to concentrate on developing new fold recognition approaches that can enhance template-based modelling for cases of analogous fold similarity.

## Materials and Methods

### Training and test sets

As a test set 150 diverse globular proteins were used (Table S1). The set spans across the whole Pfam database [32], representing a variety of folds, with each protein comprising a different Pfam domain family. All of the proteins have high quality resolved crystal structures (resolution ≤1.9 Å), are monomeric according to PISA [41], and both short (<50 residues) and very long (>270 residues) chains were excluded. Multiple sequence alignments (MSAs) were automatically generated using the jackhmmer program from the HMMER 3.0 package [http://hmmer.org].

A training subset of 10 proteins for determination of FRAGFOLD scoring parameters and benchmarking the method was selected as a representative subset of the 150 proteins dataset (Table S4). It was chosen to reasonably reflect the whole population of proteins in the full set. Selected proteins represent different folds and span different precisions achieved by PSICOV. The set was also chosen to consist of smaller proteins to make the analysis more computationally tractable.

### Contact predictions

Lists of predicted contacts used for the predictions were generated using PSICOV [20]. Briefly, the method starts by generating a covariance matrix based on the given MSA. The graphical Lasso method [42] is then applied to this matrix to find a sparse subset of the elements of a related inverse covariance matrix. By constraining the solution to be sparse it is possible to avoid overfitting of parameters to the observed data and so increase the accuracy of the final model. After a final normalisation step, the non-zero elements of the sparse inverse covariance matrix relate to pairs of columns in the MSA which are most likely to be directly coupled, and thus likely to be in contact in the native protein structure. Raw contact prediction scores produced by PSICOV were converted to [0, 1.0] probability values (*P*). The full list of PSICOV predictions is available online at http://bioinfadmin.cs.ucl.ac.uk/downloads/PSICOV/suppdata.

### Folding

FRAGFOLD [31], [43] was used to generate 3-D structures using fragment assembly. FRAGFOLD assembles folds from a mixture of supersecondary structural fragments and short fixed length fragments taken from a library of highly resolved protein structures using simulated annealing. Fragments are selected using predicted secondary structure and a threading score between the target sequence and the fragment in question. At each position in the target sequence, a shortlist of fragments that both agree with the prediction of secondary structure and which have a favourable threading energy are produced, and these lists are sampled randomly during the folding run to generate each new conformation.

Secondary structures were generated using PSIPRED [44]. The same MSAs that were used in PSICOV contact predictions served as FRAGFOLD input alignments. Duplicate rows were filtered out, and columns with gaps in the query sequence deleted and sequences with <30% sequence identity to the target were also removed. To make the simulations more representative of blind predictions, fragments with detectable sequence similarity to each target protein were removed from the standard FRAGFOLD fragment library performing sequence search of the test set proteins against FRAGFOLD fragment library.

FRAGFOLD's force field embodies pair-wise potentials determined by inverse Boltzmann equation, solvation potential, hydrogen bonding, structure compactness and steric terms [30]. All simulations were run using all-atom representations, Replica Exchange Monte Carlo to search for low energy conformations and relative weighting of the energy function terms determined by considering the standard deviations of each term across an ensemble random chain conformations for the target, as described by Jones, *et al*. [31]. The total number of annealing steps was chosen based on the size of the target protein (10,000,000 for proteins ≥120 amino acids and 5,000,000 for smaller proteins). An ensemble of 200 models per protein was generated to ensure reasonable sampling of conformational space.

Determination of how to use residue-residue contacts (RRCON) within FRAGFOLD was performed in a step-wise procedure, one aspect at a time. An additional energy term was added to the objective function and 4 features were systematically tested using a 10 protein training set (Table S4): (1) RRCON weighting to ensure optimal balance between the impact of contacts and other potentials included in FRAGFOLD (range from 0.5 to 10.0); (2) sequence separation within the contact list to be considered, to study whether short-range or long-range contacts alone improve predictions more, rather than full contact information (≥5 amino acids (aas), ≥10 aas, 5-9 aas, 10-23 aas, >23 aas); (3) threshold of accepted contacts predicted by PSICOV (PPV>0.0, PPV>0.5, top-L contacts, top-L/2 contacts); (4) contact energy function term itself, to find the correct expression (described in the next subsection). Starting values are listed as first in brackets.

### Energy function

The final energy function used by FRAGFOLD utilizes all terms embedded in previous versions of FRAGFOLD [30], [31], [43] with the addition of a contact satisfaction term:
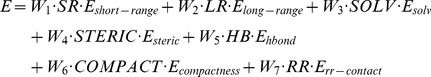
Weighting components of the potential function (W_1_ to W_7_) are determined by comparing the standard deviations of each term, across an ensemble of random conformations, to that of the short range (SR) term. Further weighting can be user-defined, and by default the STERIC terms are weighted by an additional factor of 3, RR by 5.0, while all other terms (SR, LR, SOLV, HB, COMPACT) by 1.0.

The final *E_rr-contact_* (formula below) was selected from 16 alternative functions tested. Examined functions included both terms penalizing and not penalizing non-satisfied contacts. There may be many more functions behaving in a similar way to the one presented. The selected function behaved most stably and produced best results amongst tested hypotheses. The default function (for optimization purposes) was a square well with exponential decay to *E* = 0, while the final *E_rr-contact_* is a square well function with exponential decay, expressed as:
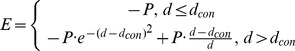
where *P* is the PSICOV contact probability (PPV), *d* is the current Cβ-Cβ distance (or Cα-Cα for glycine) and *d_con_* is the maximum contact distance predefined at *d_con_* = 8 Å. Contacts with PSICOV PPV>0 were used.

The rationale behind this function is as follows. Residues within their contact distance (0-8 Å) interact depending on their chemical nature what is described by other FRAGFOLD potentials. As residues go further apart, the attracting contact contribution diminishes exponentially up to 0. Then, depending on the initial *P* value the penalizing impact of the energy term may contribute. Non-satisfied contacts are penalized proportionally to the calculated *P* with the penalty decaying with distance to avoid generation of false positives.

### Model selection

Models were selected basing on 4 criteria:

1. best model in an ensemble (i.e. highest TM-score);
2. lowest energy model (as calculated by FRAGFOLD) denoted also as top-1 model;
3. best top-5 (i.e. highest TM-score model among 5 lowest energy models);
4. best top-5 cluster centroids (decoys were clustered on the basis of their inter-model TM-score (TMclust) or RMSD (RMSDclust); for each target, the representative models from the 5 largest clusters were selected and the highest TM-score model selected).

The first 3 model selection methods are presented in the manuscript with the most attention given to methods (2) and (3) as the most objective and suitable for blind predictions. Clustering proved to produce comparable results (often worse) to selection based purely on the basis of energy and therefore it was not included in the results in the main text (see Table S1 for results using clustering).

## Supporting Information

Figure S1 By fold comparison of best top-5 TM-score with the number of sequences in multiple sequence alignment (MSA). Red triangles – β proteins, green squares – α proteins, diamonds – α+β proteins and α/β proteins.
(TIF)

Table S1 Complete list of FRAGFOLD results.
(DOC)

Table S2 Comparison of FRAGFOLD and EVfold results.
(DOC)

Table S3 Supersecondary and fixed-length fragments fit onto experimental structures.
(DOC)

Table S4 Training subset.
(DOC)
