# Supplementary figures and images for: De Novo Structure Prediction of Globular Proteins Aided by Sequence Variation-Derived Contacts
Source: PLoS One. 2014 Mar 17;9(3):e92197. doi: 10.1371/journal.pone.0092197 (PMC3956894; doi:10.1371/journal.pone.0092197)

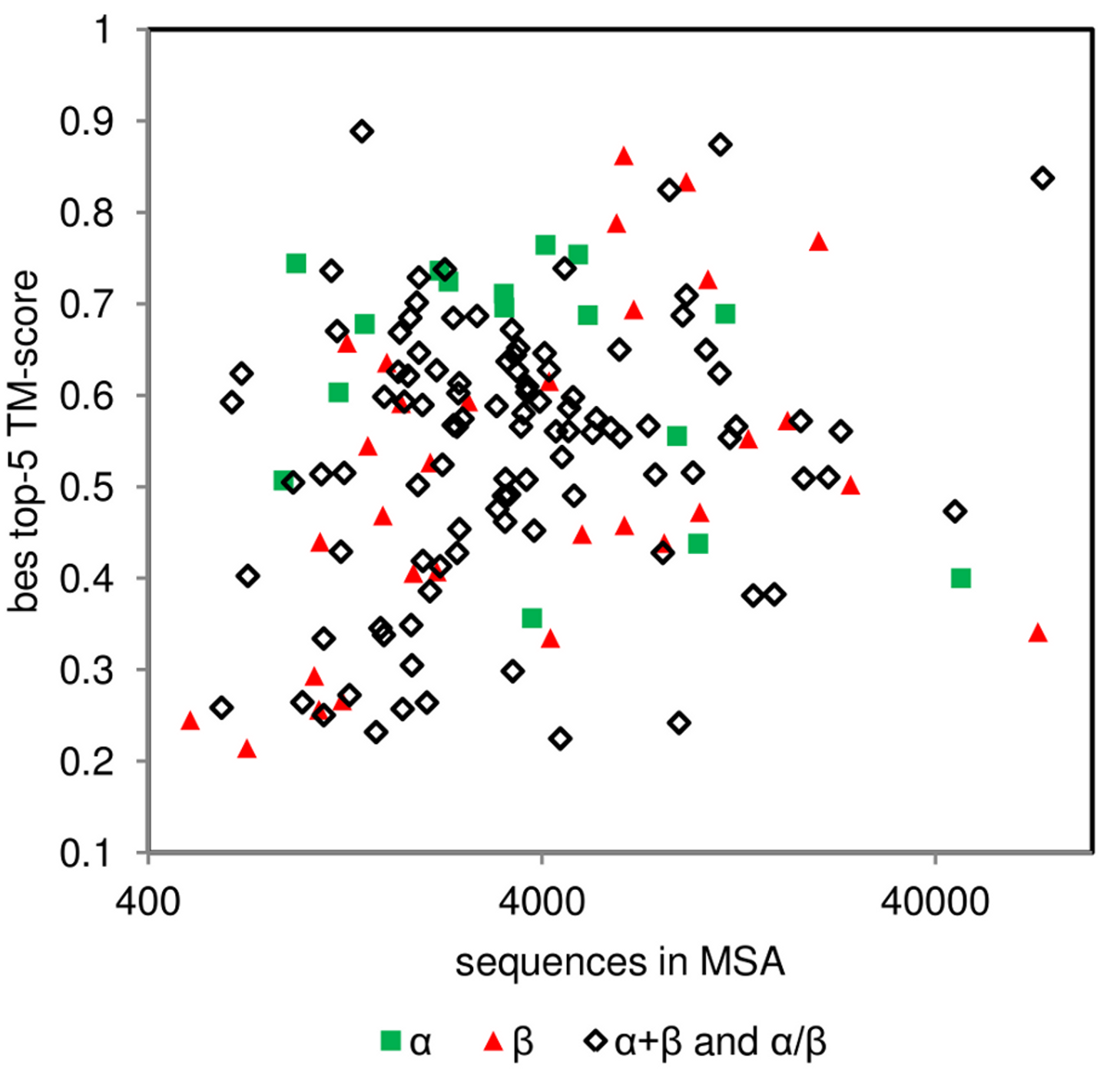

Supplement: Figure S1 — By fold comparison of best top-5 TM-score with the number of sequences in multiple sequence alignment (MSA). Red triangles – β proteins, green squares – α proteins, diamonds – α+β proteins and α/β proteins. (TIF) [file pone.0092197.s001.tif]
