# Supplementary material for: De Novo Structure Prediction of Globular Proteins Aided by Sequence Variation-Derived Contacts
Source: PLoS One. 2014 Mar 17;9(3):e92197. doi: 10.1371/journal.pone.0092197 (PMC3956894; doi:10.1371/journal.pone.0092197)
Supplement: Table S1 — Complete list of FRAGFOLD results. (DOC) [file pone.0092197.s002.doc]

**Table S1. Complete list of FRAGFOLD results.**

| protein | length | MSA size | top-L PSICOV precision | fold | MQA_LR_ contacts score^#^ | mean inter-residue TM-score | CS score^$^ | no contacts TM-score* | contacts only TM-score* | RRCON *all* contacts TM-score* | RRCON *sequential* contacts TM-score* | RRCON TMclust *all* contacts TM-score* | RRCON TMclust *sequential* contacts TM-score* | RRCON RMSDclust *all* contacts TM-score* | RRCON RMSDclust *sequential* contacts TM-score* |
| --- | --- | --- | --- | --- | --- | --- | --- | --- | --- | --- | --- | --- | --- | --- | --- |
| 1a3aA | 145 | 5378 | 0.85 | α+β | 1.76 | 0.28 | 3.72 | 0.44 | 0.53 | 0.56 | 0.56 | 0.56 | 0.54 | 0.56 | 0.54 |
| 1a6mA | 151 | 3209 | 0.35 | α | 0.20 | 0.24 | 1.91 | 0.49 | 0.57 | 0.38 | 0.70 | 0.47 | 0.77 | 0.65 | 0.51 |
| 1a70A | 97 | 4798 | 0.58 | α+β | 2.04 | 0.21 | 3.55 | 0.39 | 0.56 | 0.52 | 0.60 | 0.53 | 0.71 | 0.58 | 0.49 |
| 1aapA | 56 | 2435 | 0.86 | small protein | 1.77 | 0.21 | 3.22 | 0.25 | 0.44 | 0.43 | 0.47 | 0.46 | 0.50 | 0.39 | 0.42 |
| 1abaA | 87 | 3539 | 0.53 | α/β | 1.94 | 0.22 | 3.49 | 0.42 | 0.43 | 0.50 | 0.57 | 0.54 | 0.57 | 0.46 | 0.53 |
| 1ag6A | 99 | 1279 | 0.65 | β | 1.73 | 0.17 | 2.89 | 0.35 | 0.48 | 0.57 | 0.66 | 0.55 | 0.65 | 0.41 | 0.66 |
| 1aoeA | 192 | 2468 | 0.50 | α/β | 0.28 | 0.16 | 1.37 | 0.24 | 0.30 | 0.23 | 0.45 | 0.22 | 0.22 | 0.26 | 0.23 |
| 1atlA | 200 | 1869 | 0.43 | α+β | 0.13 | 0.21 | 1.58 | 0.31 | 0.50 | 0.30 | 0.32 | 0.50 | 0.25 | 0.49 | 0.32 |
| 1atzA | 75 | 3068 | 0.76 | α/β | 1.52 | 0.27 | 3.44 | 0.34 | 0.49 | 0.64 | 0.58 | 0.63 | 0.62 | 0.65 | 0.59 |
| 1avsA | 81 | 11693 | 0.70 | α | 1.44 | 0.48 | 4.78 | 0.47 | 0.52 | 0.69 | 0.68 | 0.61 | 0.49 | 0.58 | 0.49 |
| 1bdoA | 80 | 8179 | 0.78 | β | 1.01 | 0.18 | 2.26 | 0.21 | 0.40 | 0.44 | 0.35 | 0.44 | 0.34 | 0.40 | 0.38 |
| 1bebA | 156 | 1577 | 0.22 | β | 0.05 | 0.29 | 2.09 | 0.51 | 0.43 | 0.52 | 0.42 | 0.49 | 0.54 | 0.49 | 0.48 |
| 1behA | 184 | 1116 | 0.45 | β (cath: α+β) | 0.08 | 0.18 | 1.34 | 0.26 | 0.38 | 0.25 | 0.25 | 0.20 | 0.18 | 0.23 | 0.28 |
| 1bkrA | 108 | 1418 | 0.50 | α | 1.37 | 0.25 | 3.15 | 0.33 | 0.51 | 0.64 | 0.68 | 0.64 | 0.68 | 0.64 | 0.49 |
| 1brfA | 53 | 1395 | 0.58 | β | 1.55 | 0.93 | 8.04 | 0.89 | 0.51 | 0.89 | 0.89 | 0.88 | 0.90 | 0.88 | 0.88 |
| 1bsgA | 266 | 4688 | 0.68 | α+β | 0.06 | 0.16 | 1.17 | 0.21 | 0.41 | 0.24 | 0.59 | 0.21 | 0.59 | 0.25 | 0.59 |
| 1c44A | 123 | 1116 | 0.33 | α+β | 0.44 | 0.24 | 2.09 | 0.31 | 0.47 | 0.33 | 0.32 | 0.32 | 0.33 | 0.48 | 0.32 |
| 1c52A | 131 | 9972 | 0.45 | α | 2.27 | 0.16 | 3.37 | 0.24 | 0.43 | 0.43 | 0.44 | 0.41 | 0.36 | 0.43 | 0.32 |
| 1c9oA | 66 | 4171 | 0.67 | β | 1.77 | 0.30 | 3.90 | 0.47 | 0.54 | 0.62 | 0.65 | 0.55 | 0.57 | 0.54 | 0.56 |
| 1cc8A | 72 | 8423 | 0.72 | α+β | 2.87 | 0.52 | 6.51 | 0.61 | 0.56 | 0.78 | 0.82 | 0.72 | 0.80 | 0.67 | 0.60 |
| 1chdA | 198 | 1948 | 0.81 | α/β | 1.98 | 0.25 | 3.71 | 0.30 | 0.63 | 0.65 | 0.58 | 0.64 | 0.58 | 0.65 | 0.41 |
| 1cjwA | 166 | 21355 | 0.69 | α+β | 3.05 | 0.22 | 4.59 | 0.30 | 0.53 | 0.55 | 0.51 | 0.55 | 0.52 | 0.47 | 0.48 |
| 1ckeA | 212 | 1988 | 0.68 | α/β | 0.25 | 0.16 | 1.39 | 0.30 | 0.37 | 0.27 | 0.59 | 0.22 | 0.50 | 0.33 | 0.44 |
| 1ctfA | 68 | 1851 | 0.74 | α+β | 2.00 | 0.34 | 4.40 | 0.51 | 0.57 | 0.68 | 0.67 | 0.61 | 0.60 | 0.50 | 0.53 |
| 1cxyA | 81 | 2453 | 0.60 | α+β | 1.85 | 0.24 | 3.51 | 0.28 | 0.56 | 0.62 | 0.60 | 0.48 | 0.60 | 0.58 | 0.58 |
| 1cznA | 169 | 4497 | 0.70 | α/β | 1.82 | 0.19 | 3.15 | 0.36 | 0.49 | 0.54 | 0.53 | 0.52 | 0.54 | 0.50 | 0.49 |
| 1d0qA | 102 | 2513 | 0.73 | α+β | 1.70 | 0.23 | 3.30 | 0.26 | 0.48 | 0.54 | 0.57 | 0.51 | 0.60 | 0.57 | 0.50 |
| 1d1qA | 159 | 3593 | 0.67 | α/β | 1.59 | 0.16 | 2.74 | 0.27 | 0.45 | 0.48 | 0.58 | 0.48 | 0.57 | 0.43 | 0.38 |
| 1d4oA | 177 | 985 | 0.40 | α/β | 0.12 | 0.20 | 1.51 | 0.30 | 0.35 | 0.27 | 0.26 | 0.24 | 0.28 | 0.28 | 0.30 |
| 1dbxA | 152 | 4169 | 0.47 | α+β | 0.19 | 0.19 | 1.54 | 0.26 | 0.51 | 0.53 | 0.63 | 0.49 | 0.52 | 0.49 | 0.28 |
| 1dixA | 208 | 1770 | 0.27 | α+β | 0.08 | 0.17 | 1.24 | 0.29 | 0.24 | 0.32 | 0.25 | 0.29 | 0.25 | 0.28 | 0.25 |
| 1dlwA | 116 | 1217 | 0.48 | α | 1.72 | 0.29 | 3.77 | 0.39 | 0.62 | 0.60 | 0.64 | 0.66 | 0.51 | 0.49 | 0.51 |
| 1dmgA | 172 | 1993 | 0.50 | α/β | 0.98 | 0.16 | 2.11 | 0.21 | 0.31 | 0.27 | 0.42 | 0.24 | 0.20 | 0.27 | 0.33 |
| 1dqgA | 134 | 712 | 0.22 | β | 0.77 | 0.20 | 2.14 | 0.21 | 0.23 | 0.21 | 0.21 | 0.23 | 0.20 | 0.22 | 0.25 |
| 1dsxA | 87 | 1936 | 0.33 | α+β | 1.35 | 0.21 | 2.84 | 0.32 | 0.32 | 0.50 | 0.59 | 0.48 | 0.55 | 0.50 | 0.60 |
| 1eazA | 103 | 6842 | 0.82 | β | 1.77 | 0.50 | 5.28 | 0.50 | 0.63 | 0.71 | 0.69 | 0.61 | 0.61 | 0.61 | 0.69 |
| 1ej0A | 180 | 2040 | 0.51 | α/β | 0.49 | 0.23 | 2.12 | 0.25 | 0.53 | 0.26 | 0.26 | 0.58 | 0.30 | 0.26 | 0.32 |
| 1ej8A | 140 | 1886 | 0.33 | β | 1.18 | 0.17 | 2.38 | 0.31 | 0.30 | 0.28 | 0.41 | 0.35 | 0.37 | 0.36 | 0.36 |
| 1ek0A | 168 | 9318 | 0.68 | α/β | 2.20 | 0.26 | 4.03 | 0.35 | 0.65 | 0.68 | 0.71 | 0.69 | 0.62 | 0.68 | 0.57 |
| 1f6bA | 176 | 7760 | 0.48 | α/β | 1.74 | 0.18 | 3.02 | 0.37 | 0.51 | 0.51 | 0.51 | 0.47 | 0.30 | 0.47 | 0.44 |
| 1fcyA | 236 | 3773 | 0.22 | α | 0.15 | 0.17 | 1.37 | 0.37 | 0.42 | 0.35 | 0.36 | 0.47 | 0.32 | 0.27 | 0.40 |
| 1fk5A | 93 | 882 | 0.13 | α | 1.29 | 0.21 | 2.76 | 0.43 | 0.50 | 0.51 | 0.63 | 0.57 | 0.62 | 0.50 | 0.53 |
| 1fl0A | 164 | 4202 | 0.63 | β | 1.73 | 0.17 | 2.95 | 0.29 | 0.42 | 0.32 | 0.33 | 0.32 | 0.30 | 0.32 | 0.33 |
| 1fnaA | 91 | 20176 | 0.78 | β | 2.75 | 0.27 | 4.66 | 0.69 | 0.62 | 0.72 | 0.77 | 0.76 | 0.75 | 0.76 | 0.72 |
| 1fqtA | 109 | 6187 | 0.85 | β | 2.54 | 0.26 | 4.36 | 0.41 | 0.57 | 0.70 | 0.79 | 0.67 | 0.73 | 0.60 | 0.64 |
| 1fvgA | 192 | 3081 | 0.73 | α+β | 0.47 | 0.18 | 1.73 | 0.27 | 0.44 | 0.28 | 0.48 | 0.40 | 0.32 | 0.33 | 0.34 |
| 1fvkA | 188 | 3300 | 0.51 | α/β | 1.78 | 0.17 | 2.96 | 0.37 | 0.45 | 0.49 | 0.48 | 0.51 | 0.50 | 0.51 | 0.47 |
| 1fx2A | 112 | 4342 | 0.61 | α+β | 2.28 | 0.24 | 3.98 | 0.30 | 0.53 | 0.58 | 0.56 | 0.58 | 0.58 | 0.58 | 0.59 |
| 1g2rA | 94 | 933 | 0.55 | α+β | 1.30 | 0.22 | 2.87 | 0.33 | 0.57 | 0.50 | 0.50 | 0.45 | 0.47 | 0.51 | 0.41 |
| 1g9oA | 91 | 10561 | 0.89 | β | 2.18 | 0.27 | 4.04 | 0.46 | 0.60 | 0.74 | 0.73 | 0.62 | 0.68 | 0.61 | 0.57 |
| 1gbsA | 185 | 4453 | 0.65 | α+β | 0.00 | 0.20 | 1.38 | 0.26 | 0.54 | 0.35 | 0.26 | 0.55 | 0.58 | 0.35 | 0.25 |
| 1gmiA | 135 | 6478 | 0.69 | β | 1.80 | 0.21 | 3.26 | 0.32 | 0.44 | 0.46 | 0.42 | 0.35 | 0.30 | 0.41 | 0.33 |
| 1gmxA | 107 | 11304 | 0.74 | α/β | 2.32 | 0.24 | 3.97 | 0.39 | 0.57 | 0.59 | 0.62 | 0.61 | 0.59 | 0.59 | 0.58 |
| 1guuA | 50 | 8811 | 0.64 | α | 1.66 | 0.34 | 4.06 | 0.41 | 0.49 | 0.56 | 0.61 | 0.61 | 0.54 | 0.50 | 0.56 |
| 1gz2A | 138 | 3669 | 0.58 | α+β | 2.04 | 0.21 | 3.48 | 0.26 | 0.52 | 0.60 | 0.65 | 0.60 | 0.61 | 0.49 | 0.54 |
| 1gzcA | 239 | 1243 | 0.40 | β | 0.19 | 0.20 | 1.59 | 0.25 | 0.36 | 0.25 | 0.27 | 0.26 | 0.26 | 0.25 | 0.24 |
| 1h0pA | 182 | 6455 | 0.76 | β | 3.22 | 0.41 | 6.09 | 0.85 | 0.83 | 0.84 | 0.86 | 0.85 | 0.86 | 0.80 | 0.87 |
| 1h2eA | 207 | 7452 | 0.84 | α/β | 2.08 | 0.20 | 3.49 | 0.31 | 0.59 | 0.57 | 0.42 | 0.61 | 0.37 | 0.59 | 0.37 |
| 1h4xA | 110 | 2381 | 0.55 | α/β | 2.29 | 0.29 | 4.31 | 0.61 | 0.51 | 0.73 | 0.68 | 0.65 | 0.68 | 0.59 | 0.65 |
| 1h98A | 77 | 12004 | 0.47 | α+β | 1.55 | 0.22 | 3.05 | 0.35 | 0.45 | 0.55 | 0.55 | 0.58 | 0.57 | 0.61 | 0.53 |
| 1hdoA | 205 | 22968 | 0.86 | α/β | 2.57 | 0.20 | 3.99 | 0.25 | 0.53 | 0.56 | 0.55 | 0.48 | 0.52 | 0.51 | 0.55 |
| 1hfcA | 157 | 1587 | 0.47 | α+β | 0.92 | 0.17 | 2.14 | 0.26 | 0.29 | 0.34 | 0.28 | 0.22 | 0.23 | 0.34 | 0.25 |
| 1hh8A | 192 | 46416 | 0.70 | α | 1.97 | 0.29 | 3.99 | 0.50 | 0.36 | 0.40 | 0.48 | 0.49 | 0.56 | 0.41 | 0.60 |
| 1htwA | 158 | 1948 | 0.75 | α/β | 1.65 | 0.25 | 3.40 | 0.33 | 0.63 | 0.67 | 0.73 | 0.67 | 0.73 | 0.67 | 0.46 |
| 1hxnA | 210 | 1056 | 0.38 | β | 0.19 | 0.19 | 1.53 | 0.26 | 0.32 | 0.35 | 0.28 | 0.39 | 0.25 | 0.28 | 0.29 |
| 1i1jA | 106 | 1092 | 0.49 | β | 1.09 | 0.18 | 2.33 | 0.22 | 0.34 | 0.43 | 0.45 | 0.43 | 0.41 | 0.45 | 0.26 |
| 1i1nA | 224 | 4062 | 0.74 | α/β | 1.73 | 0.23 | 3.35 | 0.30 | 0.62 | 0.62 | 0.65 | 0.60 | 0.60 | 0.61 | 0.38 |
| 1i4jA | 110 | 2468 | 0.69 | α+β | 1.94 | 0.31 | 4.10 | 0.39 | 0.56 | 0.61 | 0.64 | 0.59 | 0.64 | 0.66 | 0.58 |
| 1i58A | 189 | 44813 | 0.54 | α+β | 1.73 | 0.18 | 3.00 | 0.34 | 0.44 | 0.41 | 0.47 | 0.39 | 0.46 | 0.49 | 0.45 |
| 1i5gA | 144 | 12455 | 0.56 | α/β | 2.21 | 0.22 | 3.74 | 0.45 | 0.50 | 0.54 | 0.57 | 0.51 | 0.57 | 0.53 | 0.57 |
| 1i71A | 83 | 1556 | 0.49 | β | 1.63 | 0.19 | 2.93 | 0.28 | 0.38 | 0.35 | 0.33 | 0.34 | 0.25 | 0.42 | 0.33 |
| 1ihzA | 136 | 2597 | 0.71 | β | 1.63 | 0.37 | 4.19 | 0.41 | 0.54 | 0.62 | 0.59 | 0.60 | 0.51 | 0.56 | 0.47 |
| 1iibA | 103 | 1167 | 0.71 | α/β | 2.31 | 0.38 | 4.99 | 0.54 | 0.65 | 0.68 | 0.74 | 0.65 | 0.67 | 0.65 | 0.61 |
| 1im5A | 179 | 5984 | 0.69 | α/β | 1.88 | 0.19 | 3.24 | 0.36 | 0.54 | 0.56 | 0.53 | 0.57 | 0.35 | 0.53 | 0.37 |
| 1iwdA | 215 | 3457 | 0.77 | α+β | 1.92 | 0.21 | 3.40 | 0.28 | 0.57 | 0.63 | 0.50 | 0.57 | 0.29 | 0.58 | 0.31 |
| 1j3aA | 129 | 2235 | 0.58 | α/β | 1.82 | 0.19 | 3.18 | 0.31 | 0.51 | 0.48 | 0.52 | 0.51 | 0.52 | 0.48 | 0.50 |
| 1jbeA | 126 | 74836 | 0.84 | α/β | 3.48 | 0.50 | 6.98 | 0.70 | 0.77 | 0.81 | 0.84 | 0.81 | 0.81 | 0.74 | 0.71 |
| 1jbkA | 189 | 8919 | 0.22 | α/β | 0.09 | 0.16 | 1.23 | 0.25 | 0.30 | 0.24 | 0.29 | 0.24 | 0.22 | 0.23 | 0.32 |
| 1jfuA | 176 | 18500 | 0.70 | α/β | 1.68 | 0.17 | 2.89 | 0.31 | 0.51 | 0.50 | 0.51 | 0.50 | 0.51 | 0.50 | 0.47 |
| 1jfxA | 217 | 1827 | 0.63 | α/β | 1.80 | 0.21 | 3.26 | 0.33 | 0.52 | 0.62 | 0.52 | 0.62 | 0.33 | 0.60 | 0.25 |
| 1jkxA | 209 | 6289 | 0.63 | α/β | 1.73 | 0.24 | 3.39 | 0.36 | 0.54 | 0.65 | 0.42 | 0.65 | 0.30 | 0.65 | 0.40 |
| 1jl1A | 152 | 9674 | 0.74 | α/β | 1.45 | 0.17 | 2.67 | 0.35 | 0.50 | 0.48 | 0.52 | 0.35 | 0.47 | 0.48 | 0.47 |
| 1jo0A | 97 | 1207 | 0.46 | α+β | 1.77 | 0.40 | 4.59 | 0.55 | 0.52 | 0.67 | 0.61 | 0.63 | 0.61 | 0.64 | 0.49 |
| 1jo8A | 58 | 9303 | 0.93 | β | 2.37 | 0.87 | 8.48 | 0.84 | 0.71 | 0.84 | 0.83 | 0.83 | 0.83 | 0.83 | 0.82 |
| 1josA | 100 | 1742 | 0.64 | α+β | 1.88 | 0.27 | 3.77 | 0.39 | 0.58 | 0.67 | 0.67 | 0.67 | 0.55 | 0.56 | 0.59 |
| 1jvwA | 160 | 4566 | 0.81 | α+β | 2.35 | 0.20 | 3.77 | 0.38 | 0.58 | 0.66 | 0.74 | 0.64 | 0.73 | 0.61 | 0.72 |
| 1jwqA | 179 | 3272 | 0.92 | α/β | 0.47 | 0.19 | 1.81 | 0.31 | 0.46 | 0.31 | 0.64 | 0.31 | 0.63 | 0.31 | 0.38 |
| 1jyhA | 155 | 1860 | 0.47 | α+β | 0.78 | 0.25 | 2.54 | 0.30 | 0.38 | 0.35 | 0.29 | 0.35 | 0.35 | 0.35 | 0.29 |
| 1k6kA | 142 | 4942 | 0.51 | α | 1.83 | 0.31 | 3.97 | 0.34 | 0.64 | 0.75 | 0.75 | 0.63 | 0.71 | 0.63 | 0.50 |
| 1k7cA | 233 | 3232 | 0.59 | α/β | 1.85 | 0.19 | 3.19 | 0.25 | 0.50 | 0.51 | 0.34 | 0.51 | 0.34 | 0.49 | 0.33 |
| 1k7jA | 206 | 3946 | 0.66 | α+β | 0.07 | 0.16 | 1.20 | 0.25 | 0.38 | 0.26 | 0.59 | 0.21 | 0.53 | 0.30 | 0.46 |
| 1kidA | 193 | 3372 | 0.48 | α/β | 0.25 | 0.24 | 1.96 | 0.29 | 0.41 | 0.30 | 0.28 | 0.34 | 0.33 | 0.29 | 0.33 |
| 1kq6A | 140 | 3203 | 0.37 | α+β | 1.23 | 0.22 | 2.73 | 0.42 | 0.39 | 0.47 | 0.49 | 0.46 | 0.50 | 0.47 | 0.51 |
| 1kqrA | 160 | 1085 | 0.18 | β | 0.00 | 0.22 | 1.54 | 0.23 | 0.26 | 0.26 | 0.23 | 0.26 | 0.24 | 0.26 | 0.25 |
| 1ktgA | 137 | 15577 | 0.53 | α+β | 1.65 | 0.16 | 2.74 | 0.31 | 0.36 | 0.37 | 0.38 | 0.32 | 0.56 | 0.32 | 0.38 |
| 1ku3A | 61 | 5232 | 0.70 | α | 2.17 | 0.46 | 5.38 | 0.54 | 0.53 | 0.68 | 0.72 | 0.66 | 0.71 | 0.75 | 0.53 |
| 1kw4A | 70 | 2315 | 0.60 | α | 1.88 | 0.70 | 6.77 | 0.68 | 0.42 | 0.72 | 0.69 | 0.69 | 0.68 | 0.66 | 0.58 |
| 1lm4A | 189 | 3223 | 0.71 | α+β | 1.52 | 0.19 | 2.85 | 0.29 | 0.40 | 0.41 | 0.46 | 0.39 | 0.44 | 0.43 | 0.40 |
| 1lo7A | 140 | 5499 | 0.69 | α+β | 1.97 | 0.28 | 3.91 | 0.30 | 0.52 | 0.59 | 0.57 | 0.57 | 0.56 | 0.55 | 0.51 |
| 1lpyA | 162 | 1100 | 0.53 | α+β /α | 2.20 | 0.23 | 3.78 | 0.39 | 0.46 | 0.51 | 0.51 | 0.56 | 0.50 | 0.54 | 0.49 |
| 1m4jA | 133 | 1234 | 0.23 | α+β | 0.24 | 0.27 | 2.13 | 0.50 | 0.46 | 0.40 | 0.43 | 0.39 | 0.42 | 0.45 | 0.39 |
| 1m8aA | 61 | 690 | 0.43 | α+β /β | 1.26 | 0.60 | 5.48 | 0.71 | 0.52 | 0.62 | 0.64 | 0.74 | 0.74 | 0.60 | 0.54 |
| 1mk0A | 97 | 652 | 0.54 | α+β | 1.19 | 0.27 | 3.05 | 0.37 | 0.46 | 0.59 | 0.58 | 0.57 | 0.50 | 0.54 | 0.48 |
| 1mugA | 165 | 1516 | 0.29 | α/β | 0.27 | 0.17 | 1.49 | 0.24 | 0.41 | 0.27 | 0.25 | 0.23 | 0.22 | 0.27 | 0.38 |
| 1nb9A | 147 | 2081 | 0.67 | β | 1.41 | 0.20 | 2.84 | 0.31 | 0.40 | 0.51 | 0.53 | 0.49 | 0.53 | 0.49 | 0.51 |
| 1ne2A | 176 | 8113 | 0.53 | α/β | 1.73 | 0.20 | 3.16 | 0.37 | 0.41 | 0.42 | 0.43 | 0.42 | 0.41 | 0.42 | 0.37 |
| 1npsA | 88 | 1614 | 0.55 | β | 1.32 | 0.24 | 2.99 | 0.29 | 0.36 | 0.64 | 0.55 | 0.56 | 0.55 | 0.44 | 0.44 |
| 1nrvA | 100 | 3414 | 0.54 | α+β | 2.05 | 0.52 | 5.71 | 0.50 | 0.54 | 0.65 | 0.64 | 0.58 | 0.61 | 0.52 | 0.61 |
| 1ny1A | 235 | 3652 | 0.75 | α/β | 2.09 | 0.16 | 3.23 | 0.27 | 0.35 | 0.51 | 0.43 | 0.51 | 0.39 | 0.26 | 0.26 |
| 1o1zA | 226 | 3669 | 0.79 | α/β | 2.48 | 0.25 | 4.26 | 0.31 | 0.55 | 0.61 | 0.43 | 0.61 | 0.41 | 0.61 | 0.43 |
| 1p90A | 123 | 1258 | 0.48 | α/β | 1.18 | 0.21 | 2.67 | 0.55 | 0.46 | 0.54 | 0.52 | 0.51 | 0.53 | 0.49 | 0.44 |
| 1pchA | 88 | 2267 | 0.73 | α+β | 2.36 | 0.27 | 4.25 | 0.48 | 0.63 | 0.68 | 0.74 | 0.65 | 0.60 | 0.65 | 0.69 |
| 1pkoA | 124 | 24318 | 0.74 | β | 1.73 | 0.16 | 2.85 | 0.27 | 0.45 | 0.38 | 0.50 | 0.27 | 0.45 | 0.42 | 0.39 |
| 1qf9A | 194 | 4668 | 0.45 | α/β | 1.42 | 0.19 | 2.71 | 0.37 | 0.47 | 0.46 | 0.56 | 0.55 | 0.47 | 0.38 | 0.36 |
| 1qjpA | 137 | 4828 | 0.75 | β | 1.51 | 0.18 | 2.76 | 0.24 | 0.50 | 0.49 | 0.32 | 0.50 | 0.25 | 0.47 | 0.27 |
| 1ql0A | 241 | 1297 | 0.53 | α+β | 0.26 | 0.19 | 1.56 | 0.28 | 0.39 | 0.25 | 0.27 | 0.37 | 0.24 | 0.30 | 0.29 |
| 1r26A | 113 | 11352 | 0.71 | α/β | 3.36 | 0.79 | 8.87 | 0.87 | 0.73 | 0.86 | 0.87 | 0.89 | 0.88 | 0.81 | 0.86 |
| 1roaA | 111 | 716 | 0.36 | α+β | 0.50 | 0.27 | 2.38 | 0.39 | 0.41 | 0.36 | 0.40 | 0.30 | 0.35 | 0.39 | 0.37 |
| 1rw1A | 114 | 2736 | 0.49 | α/β | 1.91 | 0.27 | 3.77 | 0.33 | 0.53 | 0.69 | 0.70 | 0.69 | 0.64 | 0.63 | 0.61 |
| 1rw7A | 235 | 3820 | 0.71 | α/β | 0.17 | 0.17 | 1.37 | 0.22 | 0.47 | 0.22 | 0.45 | 0.20 | 0.44 | 0.20 | 0.21 |
| 1rybA | 186 | 2204 | 0.74 | α/β | 0.17 | 0.16 | 1.32 | 0.30 | 0.45 | 0.27 | 0.41 | 0.25 | 0.40 | 0.26 | 0.28 |
| 1smxA | 87 | 1445 | 0.47 | β | 1.13 | 0.22 | 2.66 | 0.30 | 0.44 | 0.54 | 0.47 | 0.51 | 0.50 | 0.51 | 0.37 |
| 1svyA | 101 | 1726 | 0.40 | α+β | 0.98 | 0.29 | 3.04 | 0.39 | 0.44 | 0.63 | 0.53 | 0.58 | 0.49 | 0.58 | 0.50 |
| 1t8kA | 77 | 4084 | 0.68 | α | 1.97 | 0.57 | 5.95 | 0.67 | 0.59 | 0.74 | 0.76 | 0.73 | 0.79 | 0.58 | 0.63 |
| 1tifA | 76 | 1590 | 0.53 | α+β | 0.95 | 0.33 | 3.26 | 0.37 | 0.44 | 0.60 | 0.60 | 0.58 | 0.60 | 0.52 | 0.60 |
| 1tqgA | 105 | 2197 | 0.54 | α | 1.94 | 0.38 | 4.57 | 0.50 | 0.62 | 0.74 | 0.70 | 0.68 | 0.75 | 0.74 | 0.61 |
| 1tqhA | 242 | 18171 | 0.77 | α/β | 2.09 | 0.17 | 3.28 | 0.29 | 0.53 | 0.50 | 0.57 | 0.45 | 0.53 | 0.42 | 0.51 |
| 1tzvA | 141 | 3200 | 0.60 | α | 2.95 | 0.38 | 5.61 | 0.57 | 0.64 | 0.72 | 0.71 | 0.70 | 0.74 | 0.69 | 0.69 |
| 1vfyA | 67 | 2077 | 0.52 | α+β | 1.04 | 0.27 | 2.92 | 0.29 | 0.42 | 0.44 | 0.39 | 0.39 | 0.41 | 0.36 | 0.37 |
| 1vhuA | 192 | 2160 | 0.70 | α/β | 2.18 | 0.26 | 4.00 | 0.40 | 0.61 | 0.63 | 0.49 | 0.61 | 0.53 | 0.61 | 0.49 |
| 1vjkA | 87 | 1923 | 0.77 | α+β | 1.60 | 0.35 | 4.07 | 0.34 | 0.53 | 0.49 | 0.70 | 0.53 | 0.60 | 0.53 | 0.60 |
| 1vmbA | 107 | 1788 | 0.55 | α+β | 1.49 | 0.24 | 3.18 | 0.34 | 0.45 | 0.58 | 0.59 | 0.59 | 0.61 | 0.50 | 0.50 |
| 1vp6A | 133 | 10064 | 0.86 | β | 1.56 | 0.19 | 2.86 | 0.36 | 0.40 | 0.52 | 0.47 | 0.38 | 0.48 | 0.41 | 0.36 |
| 1w0hA | 200 | 6328 | 0.72 | α/β | 1.76 | 0.16 | 2.91 | 0.26 | 0.49 | 0.42 | 0.55 | 0.35 | 0.49 | 0.46 | 0.38 |
| 1whiA | 122 | 2163 | 0.66 | β | 0.82 | 0.26 | 2.62 | 0.28 | 0.38 | 0.41 | 0.30 | 0.44 | 0.25 | 0.39 | 0.30 |
| 1wjxA | 112 | 1755 | 0.71 | β | 1.46 | 0.21 | 2.92 | 0.28 | 0.52 | 0.51 | 0.59 | 0.51 | 0.53 | 0.46 | 0.58 |
| 1wkcA | 168 | 2387 | 0.67 | α/β | 1.44 | 0.19 | 2.80 | 0.27 | 0.49 | 0.57 | 0.39 | 0.57 | 0.21 | 0.54 | 0.27 |
| 1xdzA | 238 | 2432 | 0.70 | α/β | 1.87 | 0.23 | 3.48 | 0.30 | 0.58 | 0.57 | 0.40 | 0.58 | 0.27 | 0.57 | 0.44 |
| 1xffA | 238 | 9108 | 0.75 | α+β | 2.00 | 0.24 | 3.68 | 0.32 | 0.61 | 0.69 | 0.38 | 0.60 | 0.28 | 0.60 | 0.37 |
| 1xkrA | 205 | 614 | 0.20 | α+β | 0.04 | 0.18 | 1.30 | 0.29 | 0.33 | 0.24 | 0.26 | 0.29 | 0.29 | 0.26 | 0.25 |
| 2arcA | 161 | 511 | 0.33 | β | 0.10 | 0.17 | 1.29 | 0.25 | 0.28 | 0.25 | 0.24 | 0.22 | 0.24 | 0.25 | 0.25 |
| 2cuaA | 122 | 16816 | 0.70 | β | 1.95 | 0.22 | 3.51 | 0.43 | 0.46 | 0.56 | 0.57 | 0.50 | 0.57 | 0.54 | 0.52 |
| 2hs1A | 99 | 72784 | 0.39 | β | 1.05 | 0.21 | 2.53 | 0.29 | 0.31 | 0.35 | 0.31 | 0.36 | 0.26 | 0.32 | 0.28 |
| 2mhrA | 118 | 950 | 0.42 | α | 1.90 | 0.38 | 4.59 | 0.51 | 0.63 | 0.74 | 0.75 | 0.72 | 0.63 | 0.71 | 0.69 |
| 2phyA | 125 | 13767 | 0.50 | α+β | 1.38 | 0.20 | 2.78 | 0.40 | 0.37 | 0.44 | 0.38 | 0.39 | 0.39 | 0.40 | 0.38 |
| 2tpsA | 226 | 3481 | 0.67 | α/β | 1.74 | 0.23 | 3.32 | 0.39 | 0.59 | 0.65 | 0.56 | 0.61 | 0.48 | 0.63 | 0.49 |
| 2vxnA | 249 | 3355 | 0.75 | α+β | 2.04 | 0.24 | 3.74 | 0.29 | 0.54 | 0.67 | 0.32 | 0.67 | 0.24 | 0.60 | 0.32 |
| 3borA | 194 | 10450 | 0.67 | α+β | 2.17 | 0.22 | 3.72 | 0.34 | 0.55 | 0.58 | 0.65 | 0.61 | 0.62 | 0.58 | 0.40 |
| 3dqgA | 148 | 5058 | 0.76 | β | 1.54 | 0.25 | 3.29 | 0.28 | 0.39 | 0.42 | 0.45 | 0.41 | 0.45 | 0.45 | 0.40 |
| 5ptpA | 222 | 13370 | 0.82 | β | 1.83 | 0.19 | 3.14 | 0.27 | 0.52 | 0.49 | 0.55 | 0.52 | 0.35 | 0.50 | 0.38 |

^#^ top-L long-range contact satisfaction score
^$^ combined model quality assessment score
* best top-5
