# Supplementary material for: De Novo Structure Prediction of Globular Proteins Aided by Sequence Variation-Derived Contacts
Source: PLoS One. 2014 Mar 17;9(3):e92197. doi: 10.1371/journal.pone.0092197 (PMC3956894; doi:10.1371/journal.pone.0092197)
Supplement: Table S2 — Comparison of FRAGFOLD and EVfold results. (DOC) [file pone.0092197.s003.doc]

**Table S2. Comparison of FRAGFOLD and EVfold results.**

| PDB ID | Uniprot ID | EVfold TM-score | FRAGFOLD TM-score |
| --- | --- | --- | --- |
| 5p21 | RASH_HUMAN | 0.757 | **0.882** |
| 1e6k | CHEY_ECOLI | 0.674 | **0.850** |
| 1rqm | THIO_ALIAC | 0.591 | **0.801** |
| 1f21 | RNH_ECOLI | 0.572 | 0.577 |
| 3tgi | TRY2_RAT | **0.597** | 0.506 |
| 2o72 | CADH1_HUMAN | Transmembrane | |
| 2hda | YES_HUMAN | 0.413 | **0.849** |
| 1r9h | O45418_CAEEL | 0.528 | **0.905** |
| 1g2e | ELAV4_HUMAN | 0.592 | **0.796** |
| 2it6 | A8MVQ9_HUMAN | **0.532** | 0.446 |
| 1wvn | PCBP1_HUMAN | 0.346 | **0.698** |
| 1hzx | OPSD_BOVIN | Transmembrane | |
| 5pti | BPT1_BOVIN | 0.567 | 0.570 |
| 1odd | OMPR_ECOLI | 0.452 | **0.616** |
| 1bkr | SPTB2_HUMAN | 0.497 | **0.760** |

Benchmark was performed on the dataset reported in Evfold [1]. Since EVfold does not produce an energy function, best (ie. highest TM-score) results are compared. FRAGFOLD parameters were the same as in the testing dataset, with 100 structures per target generated. Since FRAGFOLD is designed for modelling globular proteins, no attempt was made to predict transmembrane targets. Results basing on similar methodology tailored to predict transmembrane proteins can be found elsewhere [2]. Results where a significant (> 0.05) difference in TM-score may be observed were highlighted.

**REFERENCES**

1. Marks DS, Colwell LJ, Sheridan R, Hopf TA, Pagnani, et al. (2011) Protein 3D structure computed from evolutionary sequence variation. PLoS ONE 6(12): e28766. doi:10.1371/journal.pone.0028766

2. Nugent T, Jones DT (2012) Accurate de novo protein structure prediction of large transmembrane protein domains using a fragment-based approach and correlated mutation analysis. Proceedings of the National Academy of Sciences of the United States of America doi:10.1073/pnas.1120036109
