# Supplementary material for: De Novo Structure Prediction of Globular Proteins Aided by Sequence Variation-Derived Contacts
Source: PLoS One. 2014 Mar 17;9(3):e92197. doi: 10.1371/journal.pone.0092197 (PMC3956894; doi:10.1371/journal.pone.0092197)
Supplement: Table S3 — Supersecondary and fixed-length fragments fit onto experimental structures. (DOC) [file pone.0092197.s004.doc]

**Table S3. Supersecondary and fixed-length fragments fit onto experimental structures.**

| protein | mean RMS distance matrix error | | | |
| --- | --- | --- | --- | --- |
|  | fixed-length fragments | fixed-length fragments ranking position | supersecondary fragments | supersecondary fragments ranking position |
| 1i1jA | 1.36 | 6 | **4.47** | 1 |
| 1i71A | 1.50 | 1 | **4.42** | 2 |
| 1bdoA | 1.32 | 9 | **4.16** | 3 |
| 1qjpA | 1.16 | 23 | **4.05** | 4 |
| 1ej8A | 1.02 | 49 | **4.04** | 5 |
| 1pkoA | 1.24 | 17 | 3.88 | 6 |
| 1gzcA | 1.15 | 24 | 3.68 | 7 |
| 1roaA | 1.05 | 45 | 3.58 | 8 |
| 1npsA | 1.24 | 16 | 3.53 | 9 |
| 1ql0A | 1.29 | 13 | 3.49 | 10 |
| 1wjxA | 1.31 | 10 | 3.48 | 11 |
| 1lm4A | 1.49 | 2 | 3.45 | 12 |
| 1ag6A | 1.11 | 34 | 3.40 | 13 |
| 1jyhA | 1.00 | 55 | 3.37 | 14 |
| 1whiA | 1.35 | 7 | 3.37 | 15 |
| 1behA | 1.30 | 12 | 3.35 | 16 |
| 1c52A | 1.00 | 54 | 3.27 | 17 |
| 1iwdA | 0.91 | 84 | 3.27 | 18 |
| 5ptpA | 0.92 | 79 | 3.26 | 19 |
| 1fl0A | 1.25 | 14 | 3.25 | 20 |
| 1fnaA | 0.87 | 94 | 3.25 | 21 |
| 3dqgA | 1.21 | 20 | 3.25 | 22 |
| 1vmbA | 0.87 | 96 | 3.19 | 23 |
| 1k7jA | 1.14 | 29 | 3.14 | 24 |
| 1dmgA | 1.39 | 4 | 3.13 | 25 |
| 1fvgA | 1.14 | 30 | 3.11 | 26 |
| 1rybA | 0.89 | 86 | 3.08 | 27 |
| 1j3aA | 0.90 | 85 | 3.07 | 28 |
| 1dbxA | 1.05 | 44 | 3.07 | 29 |
| 1nb9A | 1.08 | 38 | 3.07 | 30 |
| 2arcA | 1.04 | 47 | 3.06 | 31 |
| 1w0hA | 0.94 | 73 | 3.06 | 32 |
| 1g9oA | 0.75 | 121 | 3.04 | 33 |
| 1vjkA | 1.09 | 36 | 3.00 | 34 |
| 2hs1A | 1.38 | 5 | 2.99 | 35 |
| 1gmiA | 1.21 | 19 | 2.98 | 36 |
| 1jbkA | 0.88 | 91 | 2.98 | 37 |
| 1aoeA | 1.08 | 39 | 2.97 | 38 |
| 1jvwA | 0.78 | 113 | 2.96 | 39 |
| 1kqrA | 1.15 | 25 | 2.94 | 40 |
| 1bkrA | 0.93 | 77 | 2.91 | 41 |
| 1i4jA | 0.82 | 108 | 2.91 | 42 |
| 1lo7A | 1.02 | 50 | 2.91 | 43 |
| 1m4jA | 0.99 | 58 | 2.89 | 44 |
| 1d4oA | 1.33 | 8 | 2.88 | 45 |
| 2cuaA | 1.12 | 33 | 2.86 | 46 |
| 1smxA | 1.16 | 22 | 2.86 | 47 |
| 1dqgA | 1.31 | 11 | 2.86 | 48 |
| 1dixA | 1.00 | 56 | 2.84 | 49 |
| 1ej0A | 0.84 | 101 | 2.82 | 50 |
| 1hfcA | 1.41 | 3 | 2.82 | 51 |
| 1ne2A | 1.20 | 21 | 2.81 | 52 |
| 1t8kA | 0.64 | 132 | 2.80 | 53 |
| 1cjwA | 0.95 | 71 | 2.79 | 54 |
| 1ktgA | 0.78 | 114 | 2.79 | 55 |
| 1f6bA | 1.06 | 41 | 2.77 | 56 |
| 1cxyA | 1.15 | 26 | 2.75 | 57 |
| 1jl1A | 0.88 | 92 | 2.74 | 58 |
| 1o1zA | 0.96 | 69 | 2.74 | 59 |
| 1h2eA | 0.77 | 117 | 2.74 | 60 |
| 1k7cA | 0.98 | 62 | 2.73 | 61 |
| 1a70A | 0.79 | 110 | 2.73 | 62 |
| 1gmxA | 0.97 | 65 | 2.70 | 63 |
| 1d0qA | 1.14 | 28 | 2.70 | 64 |
| 1d1qA | 0.95 | 70 | 2.70 | 65 |
| 1p90A | 0.92 | 80 | 2.69 | 66 |
| 1c9oA | 1.12 | 31 | 2.69 | 67 |
| 1cznA | 1.03 | 48 | 2.69 | 68 |
| 1jo8A | 0.78 | 112 | 2.69 | 69 |
| 1i1nA | 0.98 | 63 | 2.68 | 70 |
| 1vp6A | 0.98 | 61 | 2.68 | 71 |
| 1kidA | 0.95 | 72 | 2.67 | 72 |
| 1xffA | 0.91 | 82 | 2.65 | 73 |
| 1qf9A | 0.57 | 145 | 2.65 | 74 |
| 2vxnA | 0.84 | 99 | 2.65 | 75 |
| 1rw7A | 0.98 | 60 | 2.65 | 76 |
| 1ckeA | 0.88 | 89 | 2.64 | 77 |
| 1a3aA | 0.87 | 95 | 2.64 | 78 |
| 1jkxA | 0.87 | 97 | 2.63 | 79 |
| 1jfuA | 0.98 | 59 | 2.62 | 80 |
| 1ihzA | 0.97 | 68 | 2.61 | 81 |
| 1hh8A | 0.62 | 137 | 2.61 | 82 |
| 1jo0A | 0.70 | 125 | 2.61 | 83 |
| 1mugA | 0.93 | 78 | 2.60 | 84 |
| 1wkcA | 1.08 | 40 | 2.60 | 85 |
| 1lpyA | 0.72 | 123 | 2.59 | 86 |
| 1xdzA | 0.86 | 98 | 2.59 | 87 |
| 1i58A | 0.79 | 111 | 2.58 | 88 |
| 3borA | 0.97 | 67 | 2.55 | 89 |
| 1ek0A | 0.69 | 128 | 2.55 | 90 |
| 1hdoA | 0.88 | 88 | 2.52 | 91 |
| 1jfxA | 1.06 | 42 | 2.51 | 92 |
| 1k6kA | 0.60 | 141 | 2.50 | 93 |
| 1c44A | 0.99 | 57 | 2.49 | 94 |
| 1tqhA | 0.70 | 126 | 2.49 | 95 |
| 1bsgA | 1.02 | 51 | 2.48 | 96 |
| 1svyA | 0.94 | 76 | 2.47 | 97 |
| 1kq6A | 0.94 | 74 | 2.46 | 98 |
| 1xkrA | 0.71 | 124 | 2.45 | 99 |
| 1i5gA | 0.92 | 81 | 2.45 | 100 |
| 1avsA | 0.41 | 149 | 2.45 | 101 |
| 1chdA | 0.94 | 75 | 2.42 | 102 |
| 2tpsA | 0.88 | 93 | 2.40 | 103 |
| 2phyA | 0.64 | 133 | 2.40 | 104 |
| 1htwA | 0.82 | 107 | 2.38 | 105 |
| 1gz2A | 1.08 | 37 | 2.37 | 106 |
| 1fk5A | 0.80 | 109 | 2.37 | 107 |
| 1ny1A | 0.82 | 105 | 2.37 | 108 |
| 1m8aA | 0.84 | 100 | 2.37 | 109 |
| 1bebA | 0.98 | 64 | 2.36 | 110 |
| 1jbeA | 0.64 | 134 | 2.36 | 111 |
| 1atlA | 1.05 | 43 | 2.36 | 112 |
| 1h0pA | 0.61 | 140 | 2.35 | 113 |
| 1pchA | 0.77 | 115 | 2.35 | 114 |
| 1tzvA | 0.63 | 136 | 2.35 | 115 |
| 1mk0A | 0.83 | 104 | 2.35 | 116 |
| 1fvkA | 0.77 | 116 | 2.34 | 117 |
| 1g2rA | 0.88 | 90 | 2.34 | 118 |
| 1jwqA | 1.12 | 32 | 2.34 | 119 |
| 1rw1A | 0.83 | 103 | 2.29 | 120 |
| 1hxnA | 1.24 | 15 | 2.29 | 121 |
| 1atzA | 1.01 | 53 | 2.28 | 122 |
| 1cc8A | 0.67 | 129 | 2.26 | 123 |
| 1vhuA | 0.91 | 83 | 2.26 | 124 |
| 1aapA | 1.14 | 27 | 2.26 | 125 |
| 1ctfA | 0.83 | 102 | 2.24 | 126 |
| 1nrvA | 0.82 | 106 | 2.23 | 127 |
| 1fcyA | 0.67 | 130 | 2.22 | 128 |
| 1abaA | 1.02 | 52 | 2.21 | 129 |
| 1h4xA | 0.76 | 119 | 2.21 | 130 |
| 1gbsA | 0.69 | 127 | 2.19 | 131 |
| 1r26A | 0.57 | 144 | 2.18 | 132 |
| 1iibA | 0.76 | 120 | 2.17 | 133 |
| 1kw4A | 0.63 | 135 | 2.16 | 134 |
| 1im5A | 1.09 | 35 | 2.14 | 135 |
| 1dsxA | 0.58 | 143 | 2.14 | 136 |
| 1eazA | 0.89 | 87 | 2.12 | 137 |
| 1guuA | 0.58 | 142 | 2.10 | 138 |
| 1fx2A | 0.62 | 138 | 2.10 | 139 |
| 1josA | 0.72 | 122 | 2.09 | 140 |
| 1fqtA | 0.97 | 66 | 2.03 | 141 |
| 1a6mA | 0.53 | 147 | 2.03 | 142 |
| 1ku3A | 0.54 | 146 | 1.92 | 143 |
| 2mhrA | 0.66 | 131 | 1.87 | 144 |
| 1tifA | 1.05 | 46 | 1.86 | 145 |
| 1tqgA | 0.45 | 148 | 1.86 | 146 |
| 1dlwA | 0.62 | 139 | 1.78 | 147 |
| 1brfA | 0.30 | 150 | 1.68 | 148 |
| 1h98A | 0.77 | 118 | 1.56 | 149 |
| 1vfyA | 1.24 | 18 | 1.33 | 150 |

Identified outliers are shown in bold (in supersecondary fragments).
Sampling problems (from Table 2) are highlighted in orange and contact-related problems in green.
