# Supplementary material for: De Novo Structure Prediction of Globular Proteins Aided by Sequence Variation-Derived Contacts
Source: PLoS One. 2014 Mar 17;9(3):e92197. doi: 10.1371/journal.pone.0092197 (PMC3956894; doi:10.1371/journal.pone.0092197)
Supplement: Table S4 — Training subset. (DOC) [file pone.0092197.s005.doc]

**Table S4. Training subset.**

| UNIPROT ID | PDB ID | fold | length | PSICOV top-L precision | PSICOV ranking position | MSA sequences | crystal resolution [Å] |
| --- | --- | --- | --- | --- | --- | --- | --- |
| TNNC2_CHICK | 1avsA | α | 81 | 0.7 | 48 | 11692 | 1.75 |
| SPTB2_HUMAN | 1bkrA | α | 108 | 0.5 | 107 | 1417 | 1.1 |
| PKHA1_HUMAN | 1eazA | β | 103 | 0.82 | 11 | 6841 | 1.4 |
| Q97S59 | 1g2rA | α/β | 94 | 0.55 | 88 | 932 | 1.35 |
| NHRF1_HUMAN | 1g9oA | β | 91 | 0.89 | 3 | 10560 | 1.5 |
| GLPE_ECOLI | 1gmxA | α/β | 107 | 0.74 | 33 | 11303 | 1.1 |
| Y065_HAEIN | 1htwA | α/β | 158 | 0.75 | 28 | 1947 | 1.7 |
| ABP1_YEAST | 1jo8A | β | 58 | 0.93 | 1 | 9302 | 1.3 |
| MOG_RAT | 1pkoA | β | 124 | 0.74 | 30 | 24317 | 1.45 |
| Q8U3C7_PYRFU | 1vjkA | α+β | 87 | 0.77 | 19 | 1922 | 1.51 |
